# IDEAS: individual level differential expression analysis for single-cell RNA-seq data

**DOI:** 10.1186/s13059-022-02605-1

**Published:** 2022-01-24

**Authors:** Mengqi Zhang, Si Liu, Zhen Miao, Fang Han, Raphael Gottardo, Wei Sun

**Affiliations:** 1 Public Health Science Division, Fred Hutchison Cancer Research Center, Seattle, USA; 2 Present Address: University of Pennsylvania, Philadelphia, 19104 USA; 3 Department of Statistics, University of Washington, Seattle, USA; 4 Biomedical Data Sciences Center, Lausanne University Hospital, Lausanne, Switzerland; 5 Department of Biostatistics, University of Washington, Seattle, USA; 6 Department of Biostatistics, University of North Carolina, Chapel Hill, USA

**Keywords:** scRNA-seq, IDEAS, Differential expression

## Abstract

We consider an increasingly popular study design where single-cell RNA-seq data are collected from multiple individuals and the question of interest is to find genes that are differentially expressed between two groups of individuals. Towards this end, we propose a statistical method named IDEAS (individual level differential expression analysis for scRNA-seq). For each gene, IDEAS summarizes its expression in each individual by a distribution and then assesses whether these individual-specific distributions are different between two groups of individuals. We apply IDEAS to assess gene expression differences of autism patients versus controls and COVID-19 patients with mild versus severe symptoms.

**Supplementary Information:**

The online version contains supplementary material available at (10.1186/s13059-022-02605-1).

## Background

Single cell RNA-seq (scRNA-seq) data provide an unprecedented high-resolution view of gene expression variation within a bulk tissue sample and thus help improve our understanding of the molecular basis of complex human diseases. For example, by comparing scRNA-seq data between cases and controls, we may identify cell-type-specific gene expression signatures that are related to disease etiology and progression [1, 2].

Early scRNA-seq studies often collect many cells from one or a few individuals and seek to compare gene expression between two groups of cells after pooling relevant cells across individuals. Several methods have been developed towards this end [3–8]. As the scRNA-seq techniques evolve from a new revolution to a standard approach, many researchers start to collect scRNA-seq data from multiple individuals, and thus differential expression (DE) testing across individuals (i.e., comparing the expression of each gene between case and controls) becomes an imperative task. The existing cell level DE methods are inappropriate for individual level DE testing. This is because the sampling space of the cell level DE methods are cells but not individuals, and a significant *p*-value asserts DE if we sample more cells from the *same* set of individuals. In contrast, when comparing gene expression between two groups of individuals, the statistical inference is whether we observe DE if we collect scRNA-seq data from more individuals.

In this paper, we assume the cells have been clustered into a few cell types if needed, and then we compare gene expression between two groups of individuals for each cell type separately. A related but different task is to compare the gene expression of two cell types across individuals. Currently, our method is not applicable to this setting. To compare gene expression across individuals, one may first estimate cell type-specific gene expression per individual by adding up the RNA-seq counts of all the cells of the same cell type. This is often known as pseudo-bulk RNA-seq data. Then, we can apply DE testing methods for bulk RNA-seq data [9, 10] to the pseudo-bulk RNA-seq data. This pseudo-bulk approach captures shift of mean expression but may miss higher-order differential expression patterns, e.g., variance changes. To fully exploit the information in scRNA-seq data, we propose a new approach that captures the cell type-specific gene expression of an individual by a probability distribution and then compare such distributions across individuals. We refer to our method as individual level differential expression analysis for ScRNA-seq data (IDEAS).

## Results

### An overview of IDEAS

**Fig. 1:**
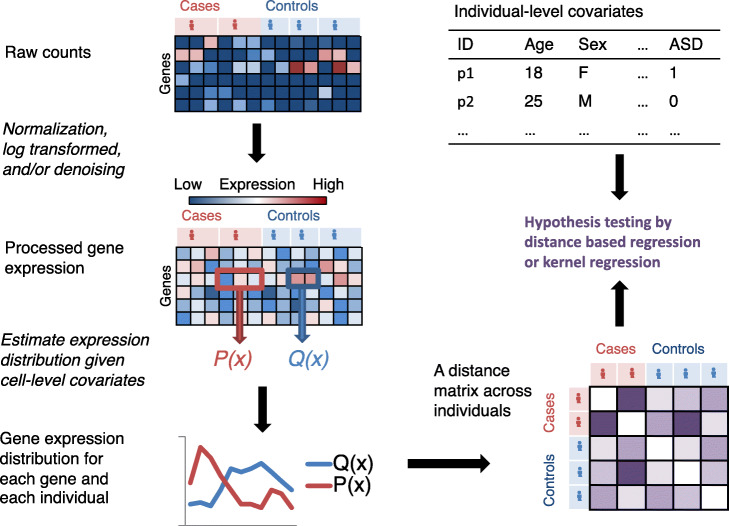
An overview of the IDEAS pipeline. Here,we illustrate a toy example with 2 cases and 3 controls, with 2 or 3 cells per individual. For each gene, we summarize its gene expression distribution within each individual (e.g., P(x) and Q(x) in the figure) and then calculate the distance of such distributions between any two individuals and obtain a distance matrix (the bottom right corner of the figure). This distance matrix, combined with additional individual-level covariates, is used for differential expression analysis between cases and controls

IDEAS performs DE testing gene by gene with respect to a categorical or continuous variable. To simplify the discussion, we consider a simple situation of two-group comparison between cases and controls for a specific gene (Fig. 1). The first step of IDEAS is to estimate the distribution of gene expression in each individual using a parametric or non-parametric method, conditioning on cell level covariates. The parametric method can be estimating a negative binomial (NB) or zero-inflated negative binomial (ZINB) distribution. The non-parametric method can be kernel density estimation (KDE) or empirical estimation of cumulative distribution function (CDF). The next step is to calculate the distance between the gene expression distributions of any two individuals by the Jensen-Shannon divergence (JSD) or Wasserstein distance (Was) [11]. The final step is to assess whether within-group distances tend to be smaller than between-group distances. We define our test statistics by a pseudo F-statistic [12], and its null distribution can be estimated by permutations. This permutation procedure is computationally efficient because we do not need to re-compute the distance matrix for each permutation. We just permute the observed distance matrix by permuting its rows and columns. When sample size is large (e.g., number of individuals >50), based on the connection between distance-based regression and kernel regression [13], we can use the asymptotic results from kernel regression to calculate *p*-values [14]. More details of IDEAS method are presented in the “Methods” section.

### Design of simulation studies

We evaluated the performance of IDEAS as well as a few other methods, including a pseudo-bulk approach (applying DESeq2 [10] on pseudo-bulk data) and four popular methods for cell level DE: Wilcoxon rank-sum test, MAST [4], scDD [5], and ZINB-WaVE [8, 15]. For cell level DE, we pooled all the cells in cases and controls separately and compare the two groups of cells. We used two versions of MAST. One is the default MAST designed for cell level DE testing and the other one is to combine MAST with a linear mixed effect model, which we refer to as MAST_lmer. The MAST_lmer approach makes an explicit assumption that the cells of one individual are more similar to each other than the cells of different individuals. We considered four versions of IDEAS methods, with two methods to estimate within-individual distributions (ZINB or KDE) and two methods to estimate distances across individuals (JSD or Was).

We simulated scRNA-seq data based on a real dataset of 62,166 cells from the prefrontal cortex (PFC) of 13 autism patients and 10 controls [1] in the following steps. First, we estimated a ZINB distribution for each gene and each cell using a data denoising neural network method called DCA (deep count autoencoder) [16]. Next, we focused on the 8626 L2/3 neuron cells to guide our simulation. We simulated the expression for 8000 genes in *n* individuals (*n*_1_ cases and *n*_2_ controls, *k* cells per individual) with one-to-one correspondence to the 8000 genes that were expressed in the highest fractions of the cells (roughly > 20% of the 8626 L2/3 neuron cells). We varies the number of individuals *n* from 10 to 40, and the number of cells *k* is set to be 360 or 1080.

For each gene, we assumed its expression followed a ZINB distribution for each cell of the *i*th individual and estimated four parameters: *μ*_*i*_ = log(mean), *ϕ*_*i*_ = log(over-dispersion), *π*_*i*_ = logit(proportion of zero-inflation), and *σ*_*i*_ (the log-transformed standard deviation of log(mean) across all the cells of the *i*th individual). The first three parameters were estimated by taking median over the cell level estimates by DCA. We estimated a multivariate normal distribution for these four parameters across the 23 individuals. Finally, we used this distribution to simulate parameters for *n* individuals and used the simulated parameters to simulate cell-level count data for each individual from a ZINB distribution. We divided the 8000 simulated genes into three groups. In the first and second 1000 genes, we added DE signal in mean value (meanDE) and variance (varDE), respectively. The remaining 6000 genes did not have any DE signal, and were referred to as equivalently expressed (EE) genes. These EE genes were used to evaluate the type I error.

### IDEAS can identify more DE patterns than the pseudo-bulk method

We first fixed the effect size of DE with 1.2-fold change for mean expression and 1.5-fold change for variance and considered eight simulation setups with four choices of sample size: 5 vs. 5, 10 vs. 10, 10 vs. 13 (to match with the sample size in the Autism data), and 20 vs. 20, and two choices of the number of cells per individual: 360 and 1080. Next, we fixed the sample size to be 10 vs. 10 and considered a series of seven effect sizes to evaluate type I error and power with respect to effect sizes. Here, we present the results for two representative simulation setups with effect sizes fixed at 1.2-fold change for mean and 1.5-fold change for variance: 5 cases vs. 5 controls with 1080 cells per individual and 13 cases vs. 10 controls with 360 cells per individual. For the former, the case with 1080 cells per individual is used since otherwise all methods have very limited power. The results of other setups can be found in Section 2 of Additional file 1.

**Fig. 2:**
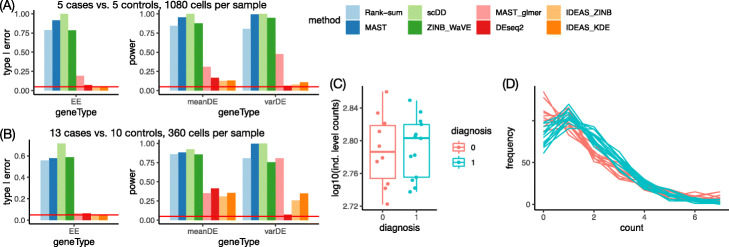
**A**, **B** Simulation results for 6,000 equivalently expressed (EE) genes, 1000 genes with DE signal in mean value, and 1000 genes with DE signal in variance in two settings. **A** Sample size (number of individuals) 5 cases versus 5 controls with 1080 cells per individual. **B** Sample size 13 cases versus 10 controls with 360 cells per individual. Here, we illustrate the results of two IDEAS methods where the distribution of gene expression within each individual was estimated by a parametric method (ZINB (zero-inflated negative binomial), kernel density estimate (KDE), and distance between distributions are quantified by the Wasserstein distance (Was)). The results of other settings of IDEAS methods are similar (Additional file 1: Fig. S1). **C** The pseudo-bulk data of 23 individuals for one gene with DE signal on variance. **D** The same gene in **C** for its empirical distribution in 23 individuals. The counts are truncated at 7 for illustration

Pooling the cells in cases and controls separately and applying cell-level DE testing methods to compare the gene expression between case cells and control cells leads to severe inflation of type I error (Fig. 2A, B). This is expected because the sample size is the number of cells, which is huge compared to the number of individuals. Interestingly, MAST combined with linear mixed effect model (MAST_lmer) only has moderate inflation of control type I error when sample size is small (5 cases vs. 5 controls, Fig. 2A) and almost no inflation of type I error when sample size is 13 cases vs. 10 controls (Fig. 2B). Since the linear mixed effect model down-weighs cell level difference, it is expected that it can achieve better type I error control when sample size is larger. We have also evaluated type I error using real data by applying different methods on permuted case/control labels and reached similar conclusions, and MAST_lmer still has noticeable inflation of type I error when sample size is 13 vs. 10 in one dataset (Additional file 1: Section 3.4) and 10 vs. 7 in the other dataset (Additional file 1: Table S6). This reflects additional features in real data that are not captured by our simulations, such as violation of gene expression distribution assumptions.

DESeq2 has slightly inflated type I error, slightly higher power than IDEAS for meanDE situation, and almost no power in the varDE situation (Fig. 2A, B). In contrast, the IDEAS methods control type I error very well and have much higher power than DESeq2 in the varDE situation (Fig. 2A-B, Additional file 1: Fig. S1). MAST_lmer has very high power to detect varDE genes, likely because the mixed effect model is suited to capture the change on variance. We also illustrate a varDE gene that shows no DE signal in pseudo-bulk data (Fig. 2C) while the difference of variation can be detected when examining the distribution of gene expression (Fig. 2D).

The results of other simulation setups, which are presented in Section 2 of Supplementary Additional file 1, support our conclusions that methods designed to compare gene expression of two groups of cells have inflated type I error and IDEAS has higher power than the pseudo-bulk + DESeq2 method. Additionally, we also demonstrated that the power of all methods increases with effect sizes (Additional file 1: Fig. S9) and slight imbalance of the sample sizes of cases and controls (13 vs. 10 compared with 10 vs. 10) does not lead to inflated type I error. Since our method uses permutations to evaluate *p*-values, severe imbalance (e.g., 3 vs. 10) will affect its accuracy since there are smaller number of possible permutations.

### NB is sufficient to capture gene expression distribution derived from UMI counts

Several recent studies have shown that a NB distribution is often sufficient to model the scRNA-seq data using UMI (unique molecular identifier)[17–20]. When applying IDEAS on simulated data, the results (the *p*-values for all the genes) using NB or ZINB distribution are highly consistent (Additional file 1: Fig. S10). Comparison of NB versus ZINB distribution using real data reaches similar conclusions (Additional file 1: Fig. S11). Therefore, by default, NB distribution is used in the following analysis. Our implementation still allows ZINB distribution, which may be useful for scRNA-seq data generated without using UMI.

### Parametric approach is more robust to the sparsity of the scRNA-seq data

**Fig. 3:**
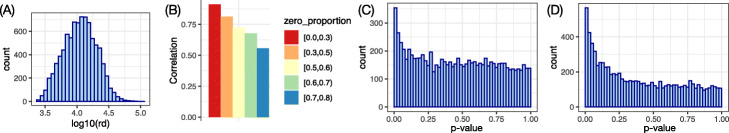
**A** Distribution of cell-level read-depth, with median around 10,000 and range from 2,414 to 105,488. **B** Correlation between the -log10(*p*-values) of DE testing between the two approaches to estimating individual-specific distribution: ZINB or empirical CDF. The genes were divided into 5 groups based on the proportion of cells where the observed gene expression is 0. **C** The *P*-value distribution when estimating individual-specific distribution using ZINB, followed by a distance calculation using the Wasserstein distance and a *p*-value calculation using permutation. **D** Same as **C** except that the input data are not the observed counts but the counts sampled from the cell specific ZINB estimated by DCA [16]

The cell level read-depth often varies considerably (Fig. 3A) and thus needs to be accounted for when estimating individual-specific distributions. Adjusting for cell level read-depth (or any other cell level covariates) is straightforward for the parametric approach. We can run a ZINB or NB regression against the log-transformed read-depth and then use the conditional distribution when setting the read-depth to certain value (e.g., the median value from all the cells across individuals). For the non-parametric approach (e.g., kernel density estimate), we can quantify read-depth effect by a linear model with log-transformed counts as the response variable and the log-transformed read-depth as a covariate. Then, read-depth adjusted gene expression can be calculated by the summation of the fitted values given median read-depth and the residuals of the linear model.

At this stage, the shortcoming of non-parametric method becomes obvious. In the real dataset, after selecting the 8260 genes that are expressed in at least 20% of 8626 L2/3 neuron cells, there are still more than half of the genes with zero expression in more than 50% of the cells. In addition, the remaining non-zero counts tend to be small, e.g., 1 to 5. A linear regression with such sparse data is highly unreliable. We illustrate this by comparing the -log10(DE *p*-value) for autism subjects versus controls obtained by two approaches: NB fit or empirical CDF fit followed by a distance calculation using the Wasserstein distance. The genes are divided into 5 categories based on the proportion of 0’s across those 8626 L2/3 neuron cells. The correlation of the two approaches decreases as the proportion of zero’s increases (Fig. 3B). After manual examination and comparison with results from pseudo bulk approaches, we conclude this is mainly due to the limitation of non-parametric approaches to handle sparse count data. Therefore, in the following analysis we focus on distribution estimation by the parametric approach (i.e., estimation of an NB distribution). When calculating the distances between individuals, using JSD or Wasserstein distance does not make much difference. We choose to focus on the Wasserstein distance due to its optimal performance in other settings [21] and recent studies of its properties [11, 22].

### Data denoising before DE testing

One angle to explain the difference between IDEAS and the pseudo-bulk method is through the bias-variance trade-off. The pseudo-bulk method summarizes the expression of many cells by summing them up, which leads to information loss (potential bias) but reduced variance. On the other hand, IDEAS tries to harvest the information from single cells at the cost of a potentially higher uncertainty in estimating the gene expression distribution across cells. One direction to improve IDEAS is via denoising the scRNA-seq data, which is a well-studied topic. A popular denoising method named DCA [16] is used in this paper. DCA estimates a ZINB distribution for each gene and each cell based on a low-dimensional space that can filter out some noise in the data.

We applied DCA to 62,166 cells of 17 cell types from the prefrontal cortex (PFC). To assess the consequence of DCA denoising, we first focused on the 8626 L2/3 neuron cells, one of the most abundant cell types. We sampled 5 counts from each cell-specific ZINB estimated by DCA and pooled them across cells to estimate an NB for each individual. We then proceeded testing using the Wasserstein distance. This new approach using DCA denoised and augmented data (Fig. 3D) has higher power than the same NB-Wasserstein approach using observed count data (Fig. 3C).

This approach to sampling counts from cell-specific ZINB estimates by DCA is flexible since we can use the sampled counts to fit an NB regression to account for any cell level covariates. However, it is also computationally intensive. An alternative approach is to directly estimate individual-level distributions by averaging the cell specific ZINBs estimated by DCA (Additional file 1: Section 1.1). This direct computation approach gives similar results to the results from NB regression (Additional file 1: Section 3.2). Therefore, we use this direct computation approach in the following analysis.

We have also studied another popular scRNA-seq denoising method named SAVER [23]. SAVER denoises scRNA-seq data by estimating a posterior mean value of a Poisson distribution for each gene and each cell while the prior is estimated by a Poisson LASSO regression, using the expression of other genes as predictors. DCA and SAVER are two representative denoising method and both perform well in a comprehensive evaluation of different denoising methods [24]. SAVER outputs the posterior Poisson distribution for each cell. We can estimate individual-level distributions by sampling from cell level distributions. Though similar to our solution for DCA-denoised data, we found directly averaging the cell specific Poisson distribution gives similar results and is computationally more efficient. Therefore we use this direct estimation approach.

### IDEAS combined with denoising improves the power to identify DE genes

**Fig. 4:**
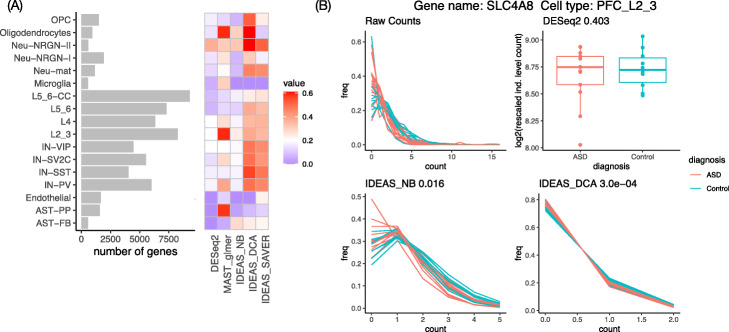
**A** The left panel shows the number of genes we studied for each cell type. A gene is included in our study if it is expressed in at least 20% of the cells. The right panel shows the estimates of the proportion of genes that are differentially expressed between autism subjects and controls for each cell type. **B** An example where IDEAS (combined with DCA) identifies strong DE signals while DESeq2 does not

We performed DE analysis between autism subjects and controls for all 17 cell types. We only considered the genes that were expressed in at least 20% of the cells for each cell type and the number of genes varied a lot across cell types: ranging from 578 (Microglia) to 9291 (L5_6-CC) (Fig. 4A). We control for multiple testing across genes using *q*-value [25]. With *q*-value cutoff at 0.2, DESeq2, IDEAS, IDEAS combined with DCA or SAVER (IDEAS_DCA or IDEAS_SAVER) identified 1134, 134, 14,774, and 7577 DE genes respectively, across the 17 cell types (Additional file 1: Table S1). These results confirm that denoising scRNA-seq data can substantially improve the power of IDEAS. An example where the DE patterns become cleaner after denoising is shown in Fig. 4B. This gene, *SLC4A8*, transports sodium and ions across cell membrane and is associated with glutamate release by neurons [26], and thus, it can have functional role in autism development. While both DCA and SAVER denoising improve power, we focus on the DCA approach in the next section to simplify discussions.

We further estimated the proportion of DE genes using a *p*-value distribution [27]. Here, we estimated the proportion of DE genes as $1 - \hat {\pi }_{0} = 1 - 2 \hat {p}_{0}(0.5)$, where $\hat {\pi }_{0}$ was the estimated proportion of non-DE genes and $\hat {p}_{0}(0.5)$ was the proportion of genes with *p*-values larger than 0.5. The DE proportion estimates were higher for excitatory neurons (e.g., layer 2/3 or layer 4 excitatory neurons) and interneurons (e.g., vasoactive intestinal polypeptide (*VIP*) and somatostatin (*SST*) expressing interneurons), partly due to relatively higher expression level in these cell types. In contrast, few DE signals were detected in astrocytes, endothelial cells, or microglia, possibly due to low gene expression in these cell types. Neu-NRGN-II was an exception where the number of expressed genes was low while all the methods identified high proportion of DE genes (Fig. 4A, Additional file 1: Table S1-2). Neurogranin (*NRGN*) is a calmodulin-binding protein, and it has been associated with Alzheimer’s disease [28] and schizophrenia [29]. Our results suggest that it is also potentially associated with autism.

### IDEAS improves the power to identify autism-related genes.

**Fig. 5:**
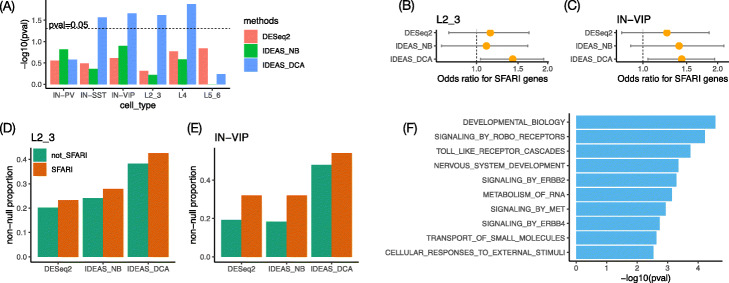
**A** Fisher’s exact test *p*-values to assess whether autism related genes (SFARI genes) have significant overlap with differential expressed genes (nominal *p*-values <0.05). **B**, **C** Odds ratios and their 95% confidence intervals derived from Fisher’s exact test in **A** for two cell types: layer 2/3 excitatory neurons (L2_3) and vasoactive intestinal polypeptide (VIP)–expressing interneurons (IN-VIP). **D**, **E** Estimates of the proportion of DE genes among those SFARI genes or non-SFARI genes for two cell types: L2_3 and IN-VIP. **F** Pathways that are over-represented by the genes that are differentially expressed between autism subjects and controls in microglia

The Simons Foundation Autism Research Initiative (SFARI) has compiled a list of autism risk genes. Most of these genes are identified because they harbor more disruptive mutations in autism subjects than in a general population. DNA mutations cannot directly affect biological function. At least part of their effect on biological systems is mediated through gene expression, and thus, these genes may be identified by DE analysis. We assessed whether there is significant overlap between cell type-specific DE genes (which are defined using a liberal *p*-value cutoff of 0.05) and SFARI genes. IDEAS combined with DCA identified significant overlaps in four cell types: excitatory neurons on layer 2/3 or layer 4, and interneurons expressing *SST* or *VIP* (Fig. 5A). In contrast, neither IDEAS nor DESeq2 identifies any significant overlap. We observe similar patterns if we just ask whether SFARI genes tend to have smaller *p*-values by gene set enrichment analysis (GSEA) (Additional file 1: Table S4). Lack of significant overlap between SFARI genes and DE genes could indicate small proportions of overlap or limited power to identify DE genes and thus that overlap is not statistically significant. For example, small proportion of overlap is the main reason for excitatory neurons on layer 2/3 (L2_3) (Fig. 5B and D) while limited power to identify DE genes is the main reason for interneurons expressing *VIP* (IN-VIP) (Fig. 5C and E).

Microglia is of particular importance for autism because it is the resident immune cells in brain and immune response is an important factor of autism etiology [30]. In our analysis, although all the methods find few DE genes in microglia, gene set enrichment analysis (GSEA) that uses the ranking of all the genes identifies a few pathways (based on the REACTOME pathway definitions) that are differentially expressed between autism subjects and controls. At adjusted *p*-value cutoff of 0.05, GSEA using the DE ranking by DESeq2 identifies one pathway: signaling by *ERBB4*. Using the ranking by IDEAS combined with DCA, GSEA identifies this pathway together with 9 others, including signaling by *ERBB2* (Additional file 1: Section 3.6). An earlier study has shown that ERBB signals can lead to proliferation and activation of microglia [31]. Separate studies have also shown that exonic deletion of *ERBB4* is associated with intellectual disability or epilepsy [32]. Our findings, combined with these earlier studies, suggest that ERBB signals may be an underlying factor that leads to different microglia activities between autism subjects and controls.

### Both denoising and non-mean-shift signals contribute to the improved power of IDEAS

We conducted additional analyses to further clarify what is driving the differentially expression signals. For example why some genes are identified as DE (differentially expressed) by IDEAS after denoising but not by DESeq2 and vice versa. Specifically, we divided the genes into four groups based on their *q*-values by DESeq2 (*q*-value cutoff 0.2) and IDEAS_DCA (*q*-value cutoff 0.1). A more liberal *q*-value cutoff was chosen for DESeq2 so that we could have enough genes in the four groups.

For each gene and each individual, IDEAS_DCA estimates an empirical distribution. We calculated the mean $\hat {\mu }$ and variance $\hat {\sigma }^{2}$ of this distribution. They are strongly correlated with each other (Additional file 1: Fig. S14). To separate the signals of variance from mean, we calculated a (pseudo) dispersion parameter: $\hat {\theta } = \hat {\mu }^{2}/(\hat {\sigma }^{2} - \hat {\mu })$ that was motivated by the mean-variance relation of a negative binomial distribution. Then, we tested whether the mean or the dispersion was associated with case/control status (Section 3.7 of Additional file 1). Among the 260 DE genes identified by IDEAS_DCA, 38/222 were identified as DE/EE (equivalently expressed) genes by DESeq2. The mean and dispersion test *p*-values of these two groups of genes are similar, suggesting the DE signals in the 222 genes are more apparent after denoising (Additional file 1: Fig. S15). When examining the proportions of genes with small mean/dispersion *p*-values, those genes identified by IDEAS_DCA but missed by DESeq2 are more likely to have DE signals only by dispersion (Additional file 1: Table S5). In addition, we observed stronger DE signals in mean value, but not in dispersion, when comparing the 93 genes identified by DESeq2 only versus the 7,907 genes identified as EE genes by both DESeq2 and IDEAS_DCA (Additional file 1: Fig. S15). This is consistent with our expectation that DESeq2 mainly detects mean-shift of gene expression. The mean DE signals of these 93 genes tend to be weaker than those 260 DE genes identified by IDEAS_DCA (by comparing the *p*-values distributions), suggesting that IDEAS_DCA misses these genes due to relatively weaker signals in mean shift.

### IDEAS identify genes/pathways related with COVID-19 severity

COVID-19 is a mild infection for most COVID patients but can be severe for a subset of them. The underlying mechanism to drive severe COVID-19 cases is an active research topic. Several recent works have revealed dysregulation of myeloid cells, particularly neutrophils. For example, by comparing scRNA-seq data from peripheral blood mononuclear cells (PBMC) of 8 mild and 10 severe COVID-19 patients, Schulte-Schrepping et al. [2] identified two neutrophil populations that are only observed in severe cases, but not in mild ones. In addition to myeloid cells, the other important compartment of immune cells is lymphoid cells, including B cells, T cells, and Nature Killer cells. In our exploration of the scRNA-seq data by Schulte-Schrepping et al., we found a large cluster of CD8+ T cells were distributed to both mild and severe COVID-19 patients, but the expression of many genes in these CD8+ T cells were different between mild and severe cases.

Similar to our analysis of the Autism data, we applied five methods to analyze the CD8+ T cells of this COVID-19 dataset: pseudo-bulk + DESeq2, MAST glmer, IDEAS, IDEAS_DCA and IDEAS_SAVER. All the IDEAS methods used the option of permutation testing, negative binomial density estimation, and Wasserstein distance. Consistent with our results from Autism data analysis, denoising substantially improved power. When controlling false discovery rate to be 5%, DESeq2, MAST_glmer, IDEAS, IDEAS_DCA, and IDEAS_SAVER identified 243, 4244, 518, 3566, and 3270 genes respectively. The large number of findings by MAST_glmer is likely due to inflated type I error since sample size is small (Additional file 1: Table S6). Next we applied GSEA analysis using REACTOME pathways. At adjusted *p*-value cutoff 0.05, GSEA identified 108, 0, 7, 20, and 12 pathways using the rankings by DESeq2, MAST_glmer, IDEAS, IDEAS_DCA, and IDEAS_SAVER respectively. It was surprising that DESeq2 identified many pathways but not many DE genes. We further performed a GSEA by ranking the genes based on their mean expression and identified 235 pathways with adjusted *p*-value cutoff 0.05. The vast majority of the pathways identified by DESeq2 (101 out of 108) and IDEAS (6 out of 7) belong to these 235 pathways, suggesting they are confounded by gene expression abundance. In contrast, only a few of the pathways identified by IDEAS_DCA (3 out of 20) or IDEAS_SAVER (1 out of 12) belong to these 235 pathways (Additional file 2).

Further examination of the GSEA results from IDEAS_DCA and IDEAS_SAVER revealed some notable pathways (Additional file 1: Fig. S16). Two *NR1H2* and *NR1H3* related pathways were identified based on IDEAS_DCA results. They are also known as the liver X receptors, *LXRA* (*NR1H3*) and *LXRB* (*NR1H2*), and function as regulators of macrophage function, lipid (e.g., cholesterol) homeostasis and inflammation. Excess levels of cholesterol can dysregulate protective immunity, for example, through LXR sumoylation in tumor microenvironment [33]. A recent genome-wide association study identified a locus in *NR1H2* associated with critically ill COVID-19 patients [34]. A *RUNX1* pathway was identified based on the results form both IDEAS_DCA and IDEAS_SAVER. *RUNX1* (runt-related transcription factor 1) is a key regulator of myeloid-derived suppressor cells [35]. A recent study suggested that *RUNX1* inhibitor may be beneficial as both a treatment and preventive therapy for COVID-19 [36]. Sumoylation related pathways were also identified based on the results from IDEAS_DCA and IDEAS_SAVER. Sumoylation is a post-translational modification process where small ubiquitin-related modifiers (*SUMO*) proteins attach to and detach from other proteins to modulate their function and it plays an important role in host immune response [37].

## Discussion

Our method IDEAS is designed for individual level DE analysis using scRNA-seq data. IDEAS compares gene expression distribution across individuals, and thus it can identify any pattern of DE including shift of mean or variance. Such flexibility is important for scRNA-seq data because of the heterogeneity of cell populations. For example, we can divide all the cells from a brain sample to excitatory neurons, interneurons, and a few glia cell types such as astrocyte, microglia, and oligodendrocyte. However, excitatory neurons and interneurons can be further divided into many smaller categories. Therefore, the DE signal may exist in a subset of the cells and IDEAS is more suitable to capture such subtle DE patterns than the pseudo-bulk method that mainly assesses shifts in mean expression.

Methods designed to assess DE across cells can be modified using a mixed effect model framework to account for cell-cell dependence within an individual and to perform DE across individuals. For example, MAST has such an option [4]. Our results show that MAST combined with linear mixed effect model can have inflated type I error when the number of individuals is small. This is because this approach captures both cell-level and individual level DE signals, and when the number of individuals is small, the cell-level variance may dominate the DE signal. Therefore, it should be used with caution when the number of individuals is small.

A key step of IDEAS is to estimate gene expression distribution for each individual. We have demonstrated that non-parametric estimates of gene expression distribution are often unreliable, especially for the genes with low expression. Therefore, we recommend parametric approaches, e.g., estimating gene expression distribution for one individual by a negative binomial distribution, which is a Poisson-Gamma mixture. In addition to the parametric or non-parametric estimates, an alternative is a semi-parametric one using a Poisson mixture with a non-parametric mixing distribution. We pursued this approach in a separate work [22].

We have observed that denoising scRNAseq data can improve the power of IDEAS. We have evaluated two representative denoising methods DCA [16] and SAVER [23]. Both methods provide estimates of cell-level gene expression distributions. Sampling from such cell level distributions is more appropriate for down-stream analysis than simply taking the posterior mean [17]. Since our method focuses on the whole distribution, it is conceptually equivalent to sampling from such cell-level distributions. Though in practice, to improve computational efficiency, we directly add up the cell level densities to derive the individual level density. A denoising method may remove both technical and biological signals; thus, it should be used with caution. In general, a denoising method works well if there is a latent structure in the data that reflects biological signals and it is not confounded with case/control status. For example, considering scRNA-seq data of many cells that can be grouped into a few cell types, then the latent structure is specified by cell type-specific gene expression. Therefore, we recommend running the denoising procedure for all the cells of different types. DCA handles read-depth difference across cells by a simple approach: dividing the observed read counts by read-depth. There is room to improve it by making more flexible correction of the read depth, for example, through a conditional variational autoencoder [38]. We will explore such more flexible denoising methods in a future work. We found both denoising and non-mean-shift signals contributed to the improved power of IDEAS. It is important to clarify what aspects of the gene expression distributions contribute to DE signals. Though due to the sparsity of the scRNA-seq data, it is hard to quantify gene expression difference in higher moments. This is an important direction for future research.

Our implementation of IDEAS is computationally efficient for large scale analysis. For example, for the analysis of cell type L2_3 of Autism data, with 8260 genes and 8626 cells from 13 cases vs. 10 controls, when running using 4 cores on a Mac with 3.8 GHz 8-Core Intel Core i7 CPU and 64GB memory, IDEAS took about 12 min to calculate the distance matrix and 16 min for testing using up to 9999 permutations. IDEAS_DCA or IDEAS_SAVER is faster than IDEAS when calculating distance matrix since they can directly use the output from DCA or SAVER to calculate individual-specific distribution function. In contrast, MAST_glmer took about 2 h, and DESeq2 took less than 1 min. Similarly, when running the simulation setup with 10 cases vs. 10 controls and 360 cells per individual, using 6 cores of a laptop with 2.3 GHz 8-Core Intel Core i9 CPU and 32GB memory, IDEAS took 7 min to calculate the distance matrix and 2 minutes for testing with up to 999 permutations. In contrast, MAST_glmer took about an hour, and DESeq2 took less than 1 min.

## Conclusions

When scRNA-seq data are collected from multiple individuals, differential expression analysis often seeks to make statistical inference for the population of individuals, e.g., cases vs. controls. We demonstrate that in such situations, methods designed to compare gene expression between cell populations often have inflated type I errors. We propose to use cell level gene expression data to estimate a distribution of gene expression for each individual, and then compare such distributions between individuals. Since we compare the distributions, our method can detect any pattern of differential expression, e.g., mean shift or variance difference. We find denoising gene expression data can improve the power of our method. In summary, our work provides a new avenue to improve the sensitivity, while preserving specificity, to detect any pattern of gene expression difference between individuals using scRNA-seq data.

## Methods

### IDEAS

#### Input and output

The input data for IDEAS include gene expression data (a matrix of scRNA-seq fragment counts per gene and per cell), the variable of interest (e.g., case-control status), together with two sets of covariates. One set is cell level covariates, such as read-depth per cell. The cell level covariates are used to estimate the gene expression distribution of each individual across all the cells. The other set is individual-level covariates such as age, gender, batch effect, etc.. The output of IDEAS is a permutation *p*-value for each gene under the null hypothesis that the expression of this gene is not associated the variable of interest, given the rest covariates.

#### Calculation of distance matrix across individuals

We examine two metrics to evaluate the distance between the gene expression distributions of two individuals. One is the Jensen-Shannon divergence (JSD) and the other one is the Wasserstein distance. For two probability distributions denoted by *P* and *Q*:

$$\textrm{JSD}(P, Q) = [D_{KL}(P \| M) + D_{KL}(Q \| M)]/2, $$

where *M* is a distribution whose density function is *f*_*M*_(*x*)=0.5[*f*_*P*_(*x*)+*f*_*Q*_(*x*)], and $D_{KL}(P \| M) = \int _{x} f_{P}(x) \log \left [f_{P}(x)/f_{M}(x)\right ] dx$ is the Kullback-Leibler divergence. The Wasserstein distance has attracted lots of attention in the machine learning fields recently [11]. We use the Wasserstein-1 distance, which is also referred to as the earth-moving distance. Intuitively, it is the minimum amount of effort to move the mass from one distribution to the other distribution. For one dimensional problem, the Wasserstein distance has a close form:

$$\text{Was}(P, Q) = \int |F_{P}(x) - F_{Q}(x)| dx, $$

where *F*_*P*_(*x*) and *F*_*Q*_(*x*) denote the cumulative distribution functions for *P* and *Q*, respectively.

We explore two approaches to estimate the distribution of gene expression across all the cells of an individual. The first approach is a parametric one where we estimate the gene expression distribution by an NB or a ZINB distribution. Here, we describe our method for the ZINB and NB is a special case for ZINB. Since our method is applied for each gene separately, we describe the procedure for one gene and ignore gene index to simplify the notation. Let *Y*_*i*_ be a random variable for gene expression of individual *i*. Then, a ZINB is a mixture distribution of a zero-inflation component and a negative binomial distribution component:

1 $$ f(Y_{i}) = \pi_{i}I(Y_{i}=0) + (1 - \pi_{i}) f_{NB}(\mu_{i}, \theta_{i}),   $$

where *π*_*i*_ is the zero-inflation proportion, *μ*_*i*_ and *θ*_*i*_ are the mean value and over-dispersion parameter for a negative binomial distribution, respectively, such that the variance of the negative binomial distribution is $\mu _{i} + \mu _{i}^{2}/\theta _{i}$.

Suppose we observe gene expression across *n*_*i*_ cells of the *i*th individual, denoted by **y**_*i*_. A naive approach is to estimate a ZINB distribution using **y**_*i*_. This approach will most likely have little power for differential expression testing because cell level read depth can vary a lot across cells/individuals, and thus, it may dominate the estimated distribution and obscure any other signals. Therefore, we perform ZINB regression (or NB regression if NB distribution is assumed) of confounding factors such as the cell level read-depth. We used function zeroinfl from R package pscl to perform the ZINB regression and used function glm.nb from R package MASS to perform NB regression.

The second approach is a non-parametric one. The specific solution depends on the distance metric used. For JSD, we estimate density using kernel method, by R function density with default Gaussian kernel. For Was, we use the R function wasserstein1d from R package transport, which takes input data points to calculate inverse of CDF and Wasserstein distance. In either case, we log transform the observed count data and then use a linear regression to obtain the adjusted log counts when all the covariates are set to their medians.

#### Data augmentation using auto-encoder

ScRNA-seq data are often noisy due to the limited number of RNA molecules per cell. Many methods have been developed to denoise scRNA-seq data. We employ two representative methods: deep count autoencoder network (DCA) [16] and SAVER [23] in our analysis. DCA exploits the low-dimensional structure of scRNA-seq data by an autoencoder, a neural network method. The input to DCA is the observed count matrix and the output is the estimated ZINB distribution for each gene and each cell. Note that this ZINB is a cell-specific distribution, and it is different from what we seek to estimate, which is an individual-specific distribution. We estimate individual-specific distributions in two ways. One is to simulate *m* counts from each cell-specific distribution and then pool them across cells to estimate the individual-specific distribution. This approach gives more flexibility to account for cell-level covariates though it is computationally intensive. The other approach is to directly add up the density estimates across cells to estimate the individual level distribution. SAVER estimates a Poisson distribution for each gene and each cell, and imposes a Gamma distribution prior for the mean value of the Poisson distribution, which is estimated by a Lasso regression using the expression of other genes as predictor. Then, the posterior mean of the Poisson distribution is estimated. Using the output from SAVER, we directly add up the Poisson density estimates across cells to estimate the individual level distribution.

#### Calculation of *p*-value

We compare two approaches to calculate a *p*-value for each gene given the distance matrix across all individuals and individual level covariates. One is a distance-based test known as Permutational Multivariate Analysis of Variance (PERMANOVA) [12, 39], and the other one is kernel based regression implemented in R package MiRKAT [14]. When the number of individuals is larger, kernel regression should have more computational advantages since it can calculate *p*-values using the asymptotic distribution of the test-statistic, though for studies with small or moderate sample sizes (number of individuals), kernel regression will also rely on permutation to assess *p*-values. For either kernel regression or PERMANOVA, the distance matrix *D* needs to be transformed to a kernel matrix **G** by

2 $$ \mathbf{G} = \left(\mathbf{I} - \frac{1}{n} \mathbf{1}\mathbf{1}'\right) \mathbf{A} \left(\mathbf{I} - \frac{1}{n} \mathbf{1}\mathbf{1}'\right),  $$

where **A**=−(1/2)**D**^2^ and **G** is the Gower’s centered matrix of **A** [12, 40]. This matrix may have some negative eigenvalues. Following earlier works, we set those negative eigenvalues to 0 [13, 41].

Let *Z* be the set of variables including the variable of interest (*X*) and all the covariates. Let **H**_*Z*_ be the hat matrix **H**_*Z*_=**Z**(**Z**^′^**Z**)^−1^**Z**^′^, and denote the trace of a square matrix **U** by *tr*(**U**). Then, the (pseudo) F statistic [12, 39] that quantifies the collective association between the gene expression and all the variables in *Z* is

3 $$ F = \frac{tr(\mathbf{H}_{Z} \mathbf{G} \mathbf{H}_{Z})}{tr[(\mathbf{I} - \mathbf{H}_{Z}) \mathbf{G} (\mathbf{I} - \mathbf{H}_{Z})]}.   $$

To generate the distribution of *F* under permutation, we permute *X* or re-sample *X* given all the covariates, following the PERMONVA-S method [42], and combine the permuted/re-sampled *X* with the covariates and generate a new data matrix *Z*_*p*_. The re-sampling approach is more desirable because it maintains the association between *X* and other covariates. However, when the number of individuals is small, it could be unstable and thus we use permutation by default. Given *Z*_*p*_, we can calculate the F-statistic following equation (3). Note that our method is different from PERMONVA-S since we consider all the covariates when calculating the F-statistics while PERMONVA-S only considers the variable of interest. When the covariates have strong association with gene expression, our method can remove their impact and thus increases the power for testing.

## Supplementary Information

**Additional file 1** Supplementary Methods and Results.

**Additional file 2** GSEA results for COVID data analysis.

**Additional file 3** Review history.

## Data Availability

The scRNA-seq data of autism patients and healthy controls, which were generated by Velmeshev et al. [1] were downloaded from https://cells.ucsc.edu/autism/rawMatrix.zip. The scRNA-seq data from COVID-19 patients, which were generated by Schulte-Schrepping et al. 2020 [2], were downloaded from https://beta.fastgenomics.org/home by searching key word *Schulte-Schrepping* in data section. The data file used in our analysis was PBMC 10x data from cohort 1. The list of SFARI ASD risk genes was downloaded from https://gene.sfari.org/database/human-gene/. These genes were scored as “syndromic” (mutations that are associated with a substantial degree of increased risk and consistently linked to additional characteristics not required for an ASD diagnosis) and/or 7 categories from 1 to 7, with high, strong, and suggestive evidence for categories 1-3. Here, we use the 350 genes that belong to the syndromic category or categories 1 to 7. Our data analysis pipeline is available at https://github.com/Sun-lab/ideas_pipeline. Our software package is available at https://github.com/Sun-lab/ideas [43]. Both repositories are licensed under the open source MIT License. The version of software package used to produce the results reported in the paper was also deposited at Zenodo [44].

## References

[1] Velmeshev D, Schirmer L, Jung D, Haeussler M, Perez Y, Mayer S, Bhaduri A, Goyal N, Rowitch DH, Kriegstein AR (2019). Single-cell genomics identifies cell type–specific molecular changes in autism. Science.

[2] Schulte-Schrepping J, Reusch N, Paclik D, Baßler K, Schlickeiser S, Zhang B, Krämer B, Krammer T, Brumhard S, Bonaguro L (2020). Severe COVID-19 is marked by a dysregulated myeloid cell compartment. Cell.

[3] Kharchenko PV, Silberstein L, Scadden DT (2014). Bayesian approach to single-cell differential expression analysis. Nat Methods.

[4] Finak G, McDavid A, Yajima M, Deng J, Gersuk V, Shalek AK, Slichter CK, Miller HW, McElrath MJ, Prlic M (2015). MAST: a flexible statistical framework for assessing transcriptional changes and characterizing heterogeneity in single-cell RNA sequencing data. Genome Biol.

[5] Korthauer KD, Chu L-F, Newton MA, Li Y, Thomson J, Stewart R, Kendziorski C (2016). A statistical approach for identifying differential distributions in single-cell RNA-seq experiments. Genome Biol.

[6] Vu TN, Wills QF, Kalari KR, Niu N, Wang L, Rantalainen M, Pawitan Y (2016). Beta-poisson model for single-cell RNA-seq data analyses. Bioinformatics.

[7] Qiu X, Hill A, Packer J, Lin D, Ma Y-A, Trapnell C (2017). Single-cell mRNA quantification and differential analysis with Census. Nat Methods.

[8] Van den Berge K, Perraudeau F, Soneson C, Love MI, Risso D, Vert J-P, Robinson MD, Dudoit S, Clement L (2018). Observation weights unlock bulk RNA-seq tools for zero inflation and single-cell applications. Genome Biol.

[9] Soneson C, Delorenzi M (2013). A comparison of methods for differential expression analysis of RNA-seq data. BMC Bioinformatics.

[10] Love MI, Huber W, Anders S (2014). Moderated estimation of fold change and dispersion for RNA-seq data with DESeq2. Genome Biol.

[11] Panaretos VM, Zemel Y (2019). Statistical aspects of Wasserstein distances. Ann Rev Stat Appl.

[12] Anderson MJ (2001). A new method for non-parametric multivariate analysis of variance. Aust Ecol.

[13] Pan W (2011). Relationship between genomic distance-based regression and kernel machine regression for multi-marker association testing. Genet Epidemiol.

[14] Wilson N, Zhao N, Zhan X, Koh H, Fu W, Chen J, Li H, Wu MC, Plantinga AM (2021). Mirkat: kernel machine regression-based global association tests for the microbiome. Bioinformatics.

[15] Risso D, Perraudeau F, Gribkova S, Dudoit S, Vert J-P (2018). A general and flexible method for signal extraction from single-cell RNA-seq data. Nat Commun.

[16] Eraslan G, Simon LM, Mircea M, Mueller NS, Theis FJ (2019). Single-cell RNA-seq denoising using a deep count autoencoder. Nat Commun.

[17] Agarwal D, Wang J, Zhang NR (2020). Data denoising and post-denoising corrections in single cell RNA sequencing. Stat Sci.

[18] Sarkar A, Stephens M (2021). Separating measurement and expression models clarifies confusion in single-cell RNA sequencing analysis. Nat Genet.

[19] Choi K, Chen Y, Skelly DA, Churchill GA (2020). Bayesian model selection reveals biological origins of zero inflation in single-cell transcriptomics. Genome Biol.

[20] Kim TH, Zhou X, Chen M (2020). Demystifying “drop-outs” in single-cell UMI data. Genome Biol.

[21] Arjovsky M, Chintala S, Bottou L. International Conference on Machine Learning, 6-11 August 2017, International Convention Centre, Sydney, Australia. In: Proceedings of the 34th International Conference on Machine Learning. PMLR: 2017. p. 214–23.

[22] Miao Z, Kong W, Vinayak RK, Sun W, Han F. Fisher-Pitman permutation tests based on nonparametric Poisson mixtures with application to single cell genomics. arXiv preprint arXiv:2106.03022. 2021:1–20.

[23] Huang M, Wang J, Torre E, Dueck H, Shaffer S, Bonasio R, Murray JI, Raj A, Li M, Zhang NR (2018). Saver: gene expression recovery for single-cell RNA sequencing. Nat Methods.

[24] Hou W, Ji Z, Ji H, Hicks SC (2020). A systematic evaluation of single-cell RNA-sequencing imputation methods. Genome Biol.

[25] Storey JD (2003). The positive false discovery rate: a Bayesian interpretation and the q-value. Ann Stat.

[26] Sinning A, Liebmann L, Kougioumtzes A, Westermann M, Bruehl C, Hübner CA (2011). Synaptic glutamate release is modulated by the na+-driven cl-/hco3- exchanger slc4a8. J Neurosci.

[27] Storey JD, Tibshirani R (2003). Statistical significance for genomewide studies. Proc Natl Acad Sci.

[28] Kester MI, Teunissen CE, Crimmins DL, Herries EM, Ladenson JH, Scheltens P, Van Der Flier WM, Morris JC, Holtzman DM, Fagan AM (2015). Neurogranin as a cerebrospinal fluid biomarker for synaptic loss in symptomatic Alzheimer disease. JAMA Neurol.

[29] Zhang Y, Gong X, Yin Z, Cui L, Yang J, Wang P, Zhou Y, Jiang X, Wei S, Wang F (2019). Association between NRGN gene polymorphism and resting-state hippocampal functional connectivity in schizophrenia. BMC Psychiatry.

[30] Prata J, Santos SG, Almeida MI, Coelho R, Barbosa MA (2017). Bridging autism spectrum disorders and schizophrenia through inflammation and biomarkers-pre-clinical and clinical investigations. J Neuroinflammation.

[31] Calvo M, Zhu N, Tsantoulas C, Ma Z, Grist J, Loeb JA, Bennett DL (2010). Neuregulin-ErbB signaling promotes microglial proliferation and chemotaxis contributing to microgliosis and pain after peripheral nerve injury. J Neurosci.

[32] Hyder Z, Van Paesschen W, Sabir A, Sansbury FH, Burke KB, Khan N, Chandler KE, Cooper NS, Wright R, McHale E (2021). ERBB4 exonic deletions on chromosome 2q34 in patients with intellectual disability or epilepsy. Eur J Hum Genet.

[33] Ma X, Bi E, Huang C, Lu Y, Xue G, Guo X, Wang A, Yang M, Qian J, Dong C (2018). Cholesterol negatively regulates IL-9–producing CD8+ T cell differentiation and antitumor activityCholesterol negatively regulates Tc9 cells. J Exp Med.

[34] Degenhardt F, Ellinghaus D, Juzenas S, Lerga-Jaso J, Wendorff M, Maya-Miles D, Uellendahl-Werth F, ElAbd H, Ruehlemann MC, Arora J, et al.New susceptibility loci for severe COVID-19 by detailed GWAS analysis in European populations. medRxiv. 2021. 2021.07.21.21260624.

[35] Zhang J, Thakuri BKC, Zhao J, Nguyen LN, Nguyen LN, Khanal S, Cao D, Dang X, Schank M, Lu Z (2021). Long noncoding RNA runxor promotes myeloid-derived suppressor cell expansion and functions via enhancing immunosuppressive molecule expressions during latent HIV infection. J Immunol.

[36] O’Hare M, Amarnani D, Whitmore HA, An M, Marino C, Ramos L, Delgado-Tirado S, Hu X, Chmielewska N, Chandrahas A (2021). Targeting runt-related transcription factor 1 prevents pulmonary fibrosis and reduces expression of severe acute respiratory syndrome coronavirus 2 host mediators. Am J Pathol.

[37] Sajeev T, Joshi G, Arya P, Mahajan V, Chaturvedi A, Mishra RK. Sumo and sumoylation pathway at the forefront of host immune response. Front Cell Dev Biol. 2021; 9. 10.3389/fcell.2021.681057. 10.3389/fcell.2021.681057 PMC8316833 34336833

[38] Huang M, Zhang Z, Zhang NR. Dimension reduction and denoising of single-cell RNA sequencing data in the presence of observed confounding variables. bioRxiv. 2020. 2020.08.03.234765.

[39] McArdle BH, Anderson MJ (2001). Fitting multivariate models to community data: a comment on distance-based redundancy analysis. Ecology.

[40] Gower JC (1966). Some distance properties of latent root and vector methods used in multivariate analysis. Biometrika.

[41] Zhao N, Chen J, Carroll IM, Ringel-Kulka T, Epstein MP, Zhou H, Zhou JJ, Ringel Y, Li H, Wu MC (2015). Testing in microbiome-profiling studies with MiRKAT, the microbiome regression-based kernel association test. Am J Human Genet.

[42] Tang Z-Z, Chen G, Alekseyenko AV (2016). PERMANOVA-S: association test for microbial community composition that accommodates confounders and multiple distances. Bioinformatics.

[43] Sun W, Zhang M, Liu S. IDEAS. GitHub. https://github.com/Sun-lab/ideas.

[44] Sun W, Zhang M, Liu S. IDEAS. 10.5281/zenodo.5808273.

