# Supplementary material for: IDEAS: individual level differential expression analysis for single-cell RNA-seq data
Source: Genome Biol. 2022 Jan 24;23:33. doi: 10.1186/s13059-022-02605-1 (PMC8784862; doi:10.1186/s13059-022-02605-1)
Supplement: Supplementary file 1 — Additional file 1 Supplementary Methods and Results. [file 13059_2022_2605_MOESM1_ESM.pdf]

Supplementary Materials for "Individual-level Differential Expression Analysis for Single Cell RNA-seq data"

December 27, 2021

Contents

1 Supplementary Methods **2**

1.1 Implementation of DCA-direct estimation method 2

2 Supplementary results from simulation study **3**

2.1 Power analysis for all possible options of IDEAS methods 3

2.2 Power analysis for additional simulation setups with 360 cells per subject 3

2.3 Power analysis for additional simulation setups with 1080 cells per subject 5

2.4 Power analysis for different effect sizes 6

3 Supplementary results from Autism data analysis **6**

3.1 Comparison of NB vs. ZINB to characterize individual-level gene expression 6

3.2 Comparison of sampling-based approach vs. DCA-direct method 8

3.3 The number of DE genes 10

3.4 Estimates of type I error 12

3.5 Overlap with SFARI genes 13

3.6 Gene set enrichment analysis (GSEA) using REACTOME pathways 14

3.7 Mean and pseudo dispersion regression 17

4 Supplementary results from COVID-19 data analysis **20**

4.1 Data processing and DE results 20

4.2 Type I error evaluation 20

4.3 GSEA using gene mean expression level 20

4.4 GSEA results 21

1 Supplementary Methods

1.1 Implementation of DCA-direct estimation method

DCA-direct method estimates the expression distribution of one gene across the cells of an individual by making use of the parameter estimates from DCA output in a straight forward manner. DCA takes the observed count matrix (rows for genes and columns for cells) as input and outputs four parameter matrices, normalized mean matrix $\bar{M}$, dispersion parameter matrix Θ, dropout probability matrix Π, and mean matrix M. Each matrix has the same dimension as that of the input count matrix, with the value at each position providing a denoised parameter estimate for the corresponding gene and cell. The difference between normalized mean matrix $\bar{M}$ and mean matrix M lies in DCA's adjustment for cell-level read-depth. To account for the factors that read-depth varies among different cells, DCA first normalizes the input raw count matrix by size factors computed based on cell-level read-depth, before proceeding to learn essential latent features and generate denoised parameter estimates. As an intermediate output of DCA for estimating the mean parameters, $\bar{M}$ is on the normalized scale. While as a final output, M is on the original scale of raw count matrix, and it is derived by multiplying the size factors back to $\bar{M}$. For DCA-direct method, we make use of normalized mean matrix $\bar{M}$ together with Θ and Π.

For the simplicity of notation, we assume that the cells belong to one specific subject occupy the first J columns of the count matrix. For one specific gene i and a cell j, let π_{ij}, $\bar{\mu}_{ij}$ and θ_{ij} denote the corresponding elements on position (i, j) of matrices Π, $\bar{M}$ and Θ respectively. The denoised distributions from DCA with normalized mean for the expression of this gene on the J cells are:

$$P_{i1} = \text{ZINB}(\pi_{i1}, \bar{\mu}_{i1}, \theta_{i1}),\ P_{i2} = \text{ZINB}(\pi_{i2}, \bar{\mu}_{i2}, \theta_{i2}),\ \ldots,\ P_{iJ} = \text{ZINB}(\pi_{iJ}, \bar{\mu}_{iJ}, \theta_{iJ}).$$

For this individual, to get an expression distribution estimate for gene i across cells, on each possible count value, we calculate the probability estimate simply by averaging the corresponding probabilities from $P_{ij}, j = 1, 2, \ldots, J$. Once we have estimated distributions for individuals, we can calculate the distance matrix either by Jensen-Shannon divergence or Wasserstein distance, and compute p-values through kernel-based association test or Permutational Multivariate Analysis of Variance.

2 Supplementary results from simulation study

2.1 Power analysis for all possible options of IDEAS methods

We have two options for each of three choices:

- methods for testing: kernel regression (KR), and Permutational Multivariate Analysis of Variance (PERMANOVA, or PS),
- methods for density estimation: zero-inflated negative binomial (zinb) or kernel density estimate (kde),
- methods for calculating distances of two distributions: Jensen-Shannon divergence (JSD) or Wasserstein distance (Was).

Here we show the performance are similar for all 8 options to run IDEAS from two simulation setups. The results are similar in other simulation setups. It worth noting that we did find in real data analysis, the non-parametric estimation of density (kde) has worse performance when it is necessary to account for cell level read-depth difference. In the following, if not otherwise specified, the IDEAS results are based on permutation test (PS) and Wasserstein distance (Was).

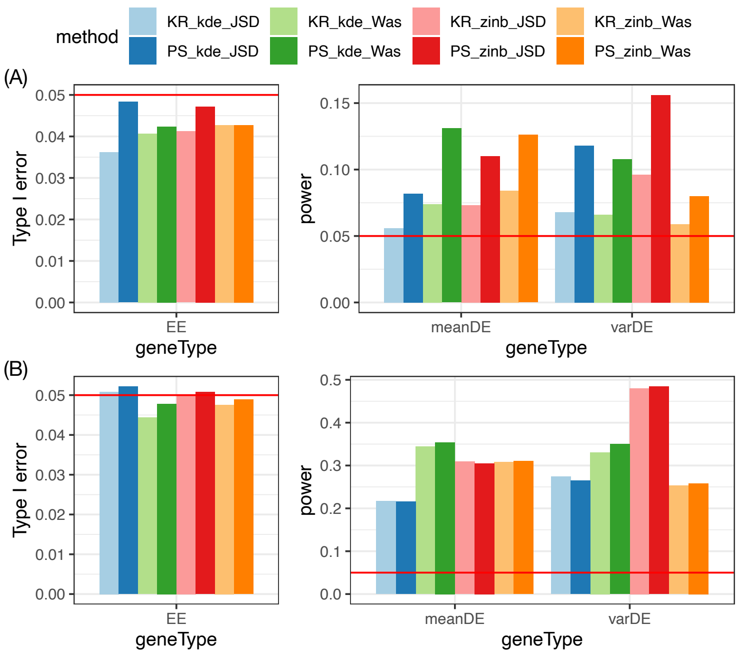

Figure S1: Evaluation of type I error and power for different versions of IDEAS methods using simulated data. (A) 5 cases vs. 5 controls, with 1080 cells per subject. (B) 13 cases vs. 10 controls, with 360 cells per subject. In the legend "KR" means kernel regression, and "PS" means Permutational Multivariate Analysis of Variance (PERMANOVA). "kde" and "zinb" are two methods to estimate the distributions by non-parametric kernel density estimates or zero-inflated negative binomial distribution, respectively. "JSD" and "was" are two methods to calculate the distance of two distributions: Jensen-Shannon divergence (JSD) or Wasserstein distance (was).

2.2 Power analysis for additional simulation setups with 360 cells per subject

Here we fix the effect sizes to be 1.2 fold change for mean expression and 1.5 fold changes for variance, and compare the results with different sample sizes. The case with sample size of 13 cases vs. 10 controls is

included in Figure 2B of main text. The IDEAS results are based on permutation test (PS) and Wasserstein distance (Was), and two options to calculate density, parametric (ZINB) or non-parametric density estimation (KDE).

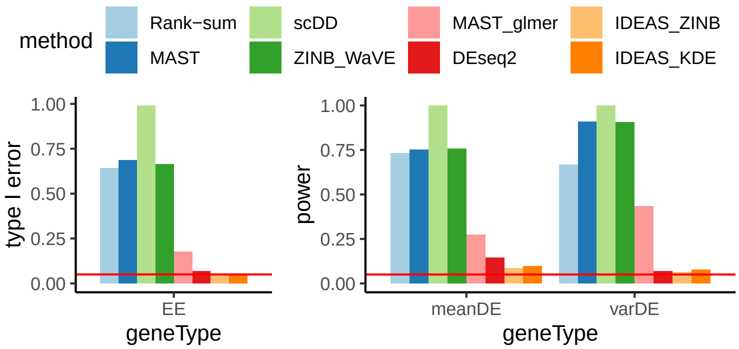

Figure S2: 5 cases vs. 5 controls.

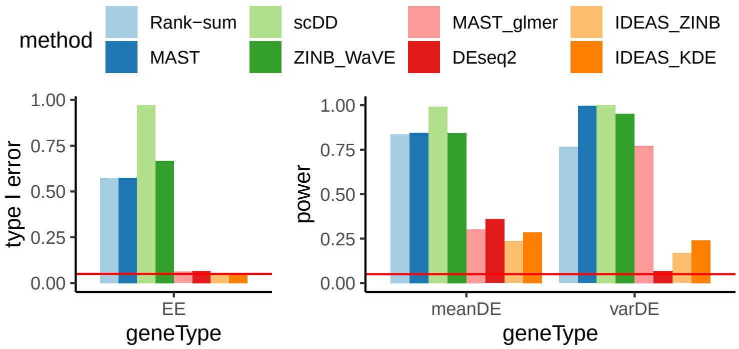

Figure S3: 10 cases vs. 10 controls.

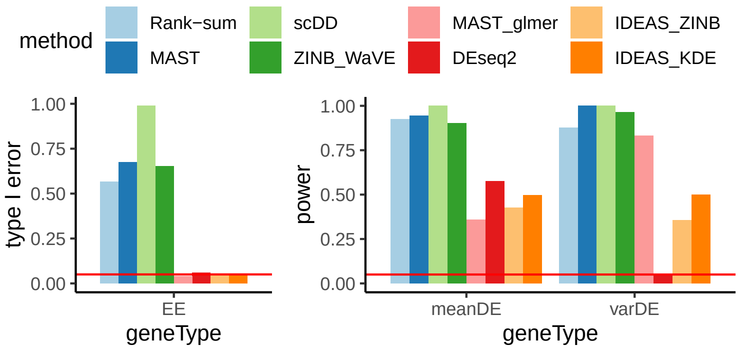

Figure S4: 20 cases vs. 20 controls.

2.3 Power analysis for additional simulation setups with 1080 cells per subject

Again, we fix the effect sizes to be 1.2 fold change for mean expression and 1.5 fold changes for variance, and compare the results with different sample sizes. The case with sample size of 5 cases vs. 5 controls is included in Figure 2A of main text. The IDEAS results are based on permutation test (PS) and Wasserstein distance (Was), and two options to calculate density, parametric (ZINB) or non-parametric density estimation (KDE). It is computationally much more demanding to run scDD and ZINB-WaVE when there are 1080 cells per subject. Since we have demonstrated that they have inflated type I error, we skip their results here.

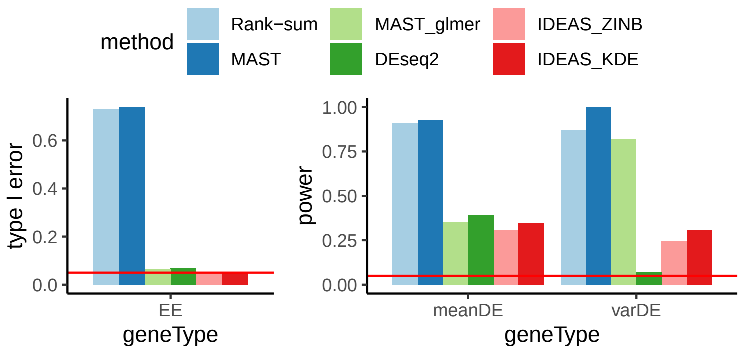

Figure S5: 10 cases vs. 10 controls.

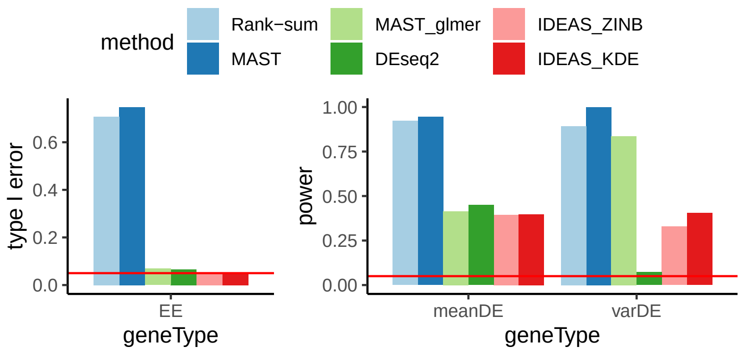

Figure S6: 13 cases vs. 10 controls.

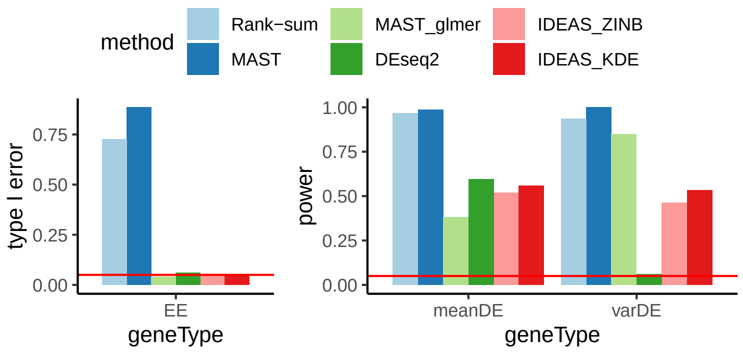

Figure S7: 20 cases vs. 20 controls.

2.4 Power analysis for different effect sizes

Here we fix the sample size to be 10 cases vs. 10 controls and examine the power for different effect sizes. The methods that compare the gene expression between two groups of cells (e.g., rank-sum test, MAST) are not included because they cannot control type I error. The IDEAS results are based on permutation test (PS) and Wasserstein distance (Was), and two options to calculate density, parametric (ZINB) or non-parametric density estimation (KDE). As we have pointed out in the main text, although MAST_glmer has good type I error control in simulations, it dose have inflated type I error in real data analysis with similar sample sizes, likely due to extra noise in the real data. Therefore MAST_glmer should be used with caution in real data analysis. For example, to double check its results after permuting case/control labels.

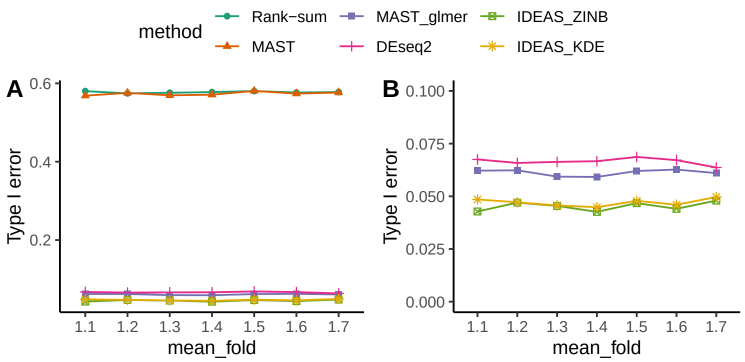

Figure S8: Compare the type I error of different methods with respect to effect size changes. The x-axis shows the fold change for mean, and the variance fold change is always mean fold change + 0.3. Note that the type I error were evaluated on the subset of equivalently expressed (EE) genes and thus their expression are not affected by neither the mean nor the variance fold change.

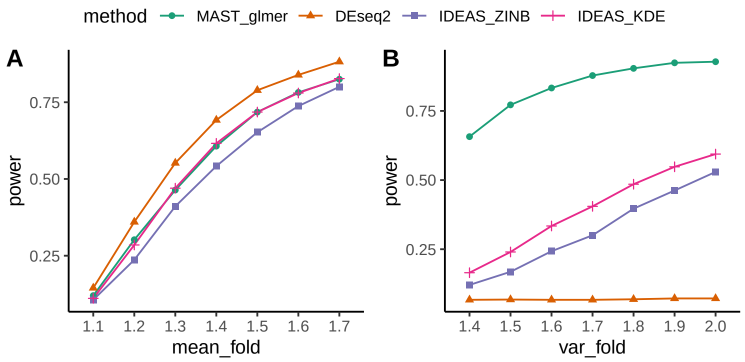

Figure S9: Compare powers of IDEAS and pseudo-bulk + DESeq2.

3 Supplementary results from Autism data analysis

3.1 Comparison of NB vs. ZINB to characterize individual-level gene expression

In this section we compare p-values from using NB or ZINB distributions to characterize individual-level gene expression through running analysis on both simulated data and real count data. Supplementary Figure S10 demonstrates the comparison on simulated data generated based on count data from the cell type excitatory neurons on layer 2/3 (L2/3) according to the process described in "Design of simulation studies" subsection in main text. The p-values from these two approaches are very similar.

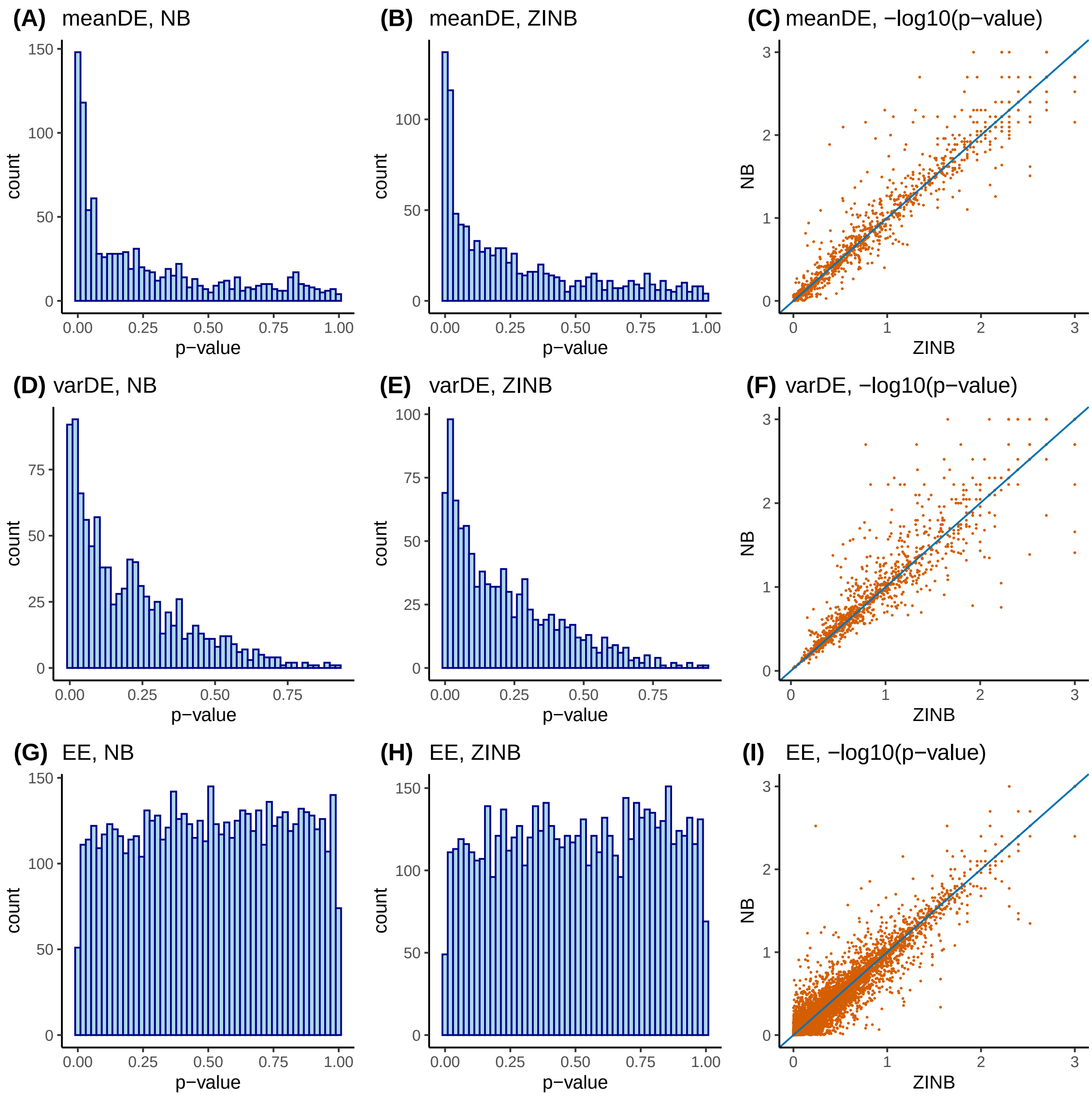

Figure S10: Comparison between the pvalues from NB and those from ZINB approaches on simulated data. Data generation process is described in "Design of simulation studies" subsection under the Result section of the main text. Each row corresponds to one group of simulated genes, with top, middle and bottom row corresponding to `meanDE`, `varDE` and `EE` respectively. The first column gives the histogram of p-values from using NB distribution in each group, and the second column gives those from using ZINB distribution. The last column provides the scatter plot comparing NB and ZINB approaches on their negative log10-transformed p-values. Each point in the scatter plot corresponds to one of the 8,000 simulated genes. For both NB and ZINB, the cell-level covariate to adjust for is read-depth, the method used to calculated distance between gene expression distribution is Wasserstein distance (Was). The p-values are computed through PERMANOVA.

Supplementary Figure S11 compares the p-values from IDEAS with NB and ZINB distributions on real count data from the cell type L2/3. The shape of the histograms and the scatter plot show that the results from these two approaches are highly consistent.

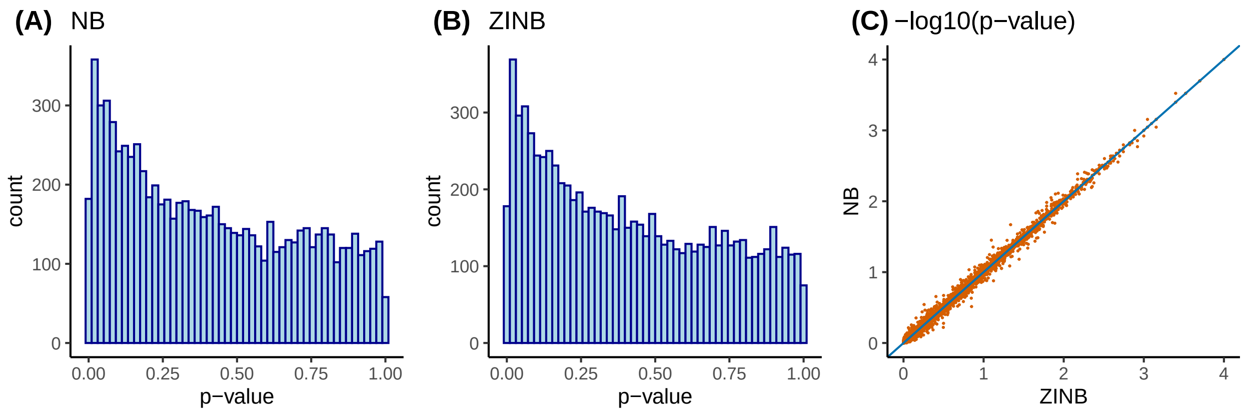

Figure S11: **(A-B)** Histogram of p-values from NB or ZINB approach on real data of cell type L2/3. **(C)** Scatter plot of -log10(p-values) from NB v.s. that from ZINB approach. Each point in the plot corresponds to one of the 8,260 genes expressed in at least 20% of 8,626 L2/3 neuron cells. The value on x-axis gives the negative log-transformed p-value with based 10 from ZINB, and the value on y-axis gives that from NB. For both NB and ZINB, the cell-level covariate to adjust for is read-depth, the method used to calculated distance between gene expression distribution is Wasserstein distance (Was), and the p-values are computed through PERMANOVA.

3.2 Comparison of sampling-based approach vs. DCA-direct method

Besides DCA-direct method, another way to utilize DCA outputs is to generate count data according to the denoised distribution estimate given by DCA for each gene and each cell, then pool the data across cells and fit a negative distribution for each individual using the pooled data, calculate the distance matrix and compute p values. Following the notations as in the subsection "Implementation of DCA-direct estimation method" in main text, different from DCA-direct method, this DCA sampling-based approach makes use of the mean matrix M instead of the normalized mean matrix $\bar{M}$, together with the dispersion parameter matrix Θ and dropout probability matrix Π.

Again for the simplicity of notation, we assume that the cells belonging to a specific individual occupy the first J columns of the count matrix. For gene i and cell j, let π_{ij}, μ_{ij} and θ_{ij} denote the corresponding elements on position (i, j) of matrices Π, M and Θ respectively. The denoised distributions from DCA with mean on the scale of original raw count for this gene on the J cells are:

$$Q_{i1} = \mathrm{ZINB}(\pi_{i1}, \mu_{i1}, \theta_{i1}),\ Q_{i2} = \mathrm{ZINB}(\pi_{i2}, \mu_{i2}, \theta_{i2}),\ \ldots,\ Q_{iJ} = \mathrm{ZINB}(\pi_{iJ}, \mu_{iJ}, \theta_{iJ}).$$

Then for each gene and each cell, we can generate multiple counts (for example, $m = 5$, 10, or 20) from the corresponding denoised distribution:

$$\begin{aligned} c_{i11}, c_{i12}, \ldots, c_{i1m} &\sim Q_{i1}, \\ c_{i21}, c_{i22}, \ldots, c_{i2m} &\sim Q_{i2}, \\ \cdots \quad & \quad \cdots \\ c_{iJ1}, c_{iJ2}, \ldots, c_{iJm} &\sim Q_{iJ}. \end{aligned}$$

Now for this individual and gene i, we have in total mJ counts sampled from the denoised distributions for the original J cells. Next, we treat all these mJ sampled counts as counts from different cells, and apply IDEAS on the matrix consisting of these sampled counts.

Supplementary Figure S12 compares the p-values from DCA sampling-based and DCA-direct approaches, under generated sample size $m = 5, 10, 20$ for DCA sampling-based approach. The trends look relatively consistent, but the DCA sampling-based approach is computationally much more expensive due to the extra computational cost to fit NB distributions.

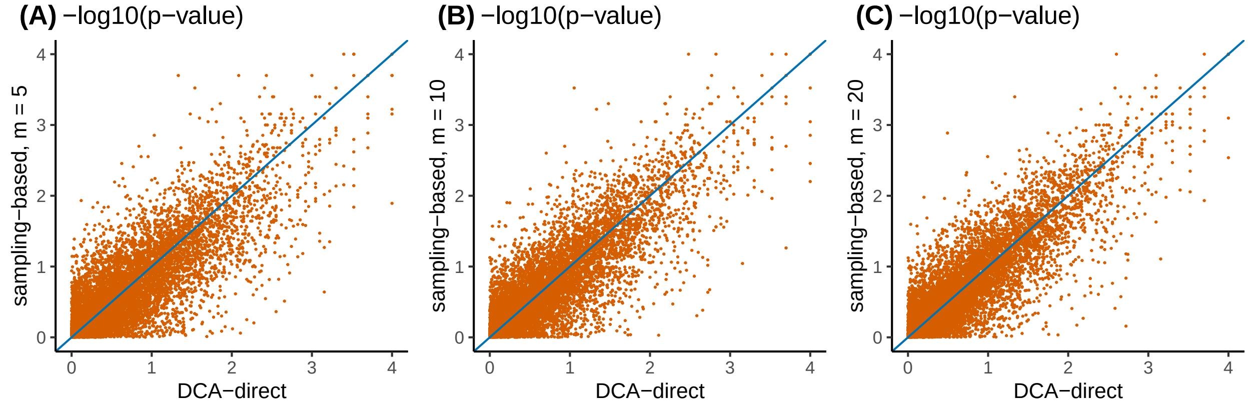

Figure S12: Scatter plots of -log10(p-values) from DCA sampling-based and DCA-direct approaches. Both approaches rely on the cell-level expression distributions estimated by DCA. The major difference is DCA sampling-based approach first sample cell-level counts and then estimate individual-level distributions, while DCA-direct approach directly estimates individual-level distributions by averaging the cell-level distributions. The cell type here is L2/3. **(A)** Comparison between -log10(p-values) from DCA sampling-based approach (y-axis) with $m = 5$ and those from DCA-direct approach (x-axis). **(B-C)** Similar comparison to that in (A), except that (B) has $m = 10$ and (C) has $m = 20$ for DCA sampling-based approach on y-axis. For DCA sampling-based approach, we fit a NB distribution per individual, with adjustment for the cell-level read-depth. For both DCA sampling-based and DCA-direct approaches, the method taken to calculated distance between gene expression distribution is Wasserstein distance (Was), and the p-values are computed through PERMANOVA.

3.3 The number of DE genes

Table S1: The number of DE genes identified by different methods using FDR 0.2 as cutoff.

Cell Type	DESeq2	IDEAS_NB	IDEAS_DCA	MAST_glmer	IDEAS_SAVER
AST-FB	0	5	0	3	0
AST-PP	13	0	0	685	0
Endothelial	1	0	0	5	1
IN-PV	151	3	3014	1123	2218
IN-SST	144	11	2453	12	1725
IN-SV2C	43	0	2806	379	1520
IN-VIP	203	10	2264	97	1233
L2_3	131	0	1784	4891	0
L4	114	0	403	17	0
L5_6	3	0	0	20	0
L5_6-CC	23	0	86	33	0
Microglia	0	0	0	57	0
Neu-mat	28	1	469	29	388
Neu-NRGN-I	82	29	0	250	131
Neu-NRGN-II	161	74	367	145	361
Oligodendrocytes	32	0	572	519	0
OPC	5	1	556	1	0
Total	1134	134	14774	8266	7577

Table S2: Estimates of the proportion of DE genes

Cell Type	Number of genes	DESeq2	MAST_glmer	IDEAS_NB	IDEAS_DCA	IDEAS_SAVER
AST-FB	584	0.00	0.05	0.28	0.24	0.22
AST-PP	1579	0.00	0.58	0.03	0.08	0.12
Endothelial	1665	0.00	0.12	0.00	0.00	0.23
IN-PV	6010	0.18	0.39	0.17	0.49	0.45
IN-SST	4049	0.17	0.17	0.18	0.56	0.48
IN-SV2C	5555	0.14	0.28	0.19	0.49	0.42
IN-VIP	4470	0.20	0.20	0.19	0.48	0.44
L2_3	8260	0.20	0.59	0.24	0.39	0.35
L4	6332	0.20	0.27	0.18	0.35	0.36
L5_6	7313	0.07	0.13	0.12	0.38	0.34
L5_6-CC	9291	0.08	0.14	0.17	0.24	0.26
Microglia	578	0.06	0.30	0.00	0.00	0.00
Neu-mat	1154	0.13	0.21	0.08	0.46	0.44
Neu-NRGN-I	1930	0.15	0.26	0.07	0.31	0.17
Neu-NRGN-II	593	0.37	0.31	0.32	0.61	0.52
Oligodendrocytes	939	0.15	0.59	0.30	0.61	0.11
OPC	1490	0.17	0.13	0.07	0.52	0.24

3.4 Estimates of type I error

We estimate type I error in real data by applying different methods on permutated case/control labels. We estimate the type I error for each method across 17 cell types by calculating the proportion of genes with p-values smaller than a threshold and summarize them by a box-plot (Supplementary Figure S13). The conclusions are consistent with our results from simulation studies. First, there are severe inflation of type I error for cell-level DE methods (rank sum test or MAST). Second, MAST_lmer has moderate level of inflation of type I error. Third, methods designed for individual level DE (DESeq2 or IDEAS methods) do not have any inflation of type I error. If case/control status indeed affects gene expression, using the permutated case/control labels leads to an mis-specified model where one informative covariate is ignored, which can leads to over-estimate of variation and thus deflated type I error. This may explain the slight deflation of type I error by IDEAS. IDEAS-DCA and IDEAS-SAVER have slightly more deflated type I error, which is likely because stronger dependence across genes are introduced after denoising the scRNA-seq data.

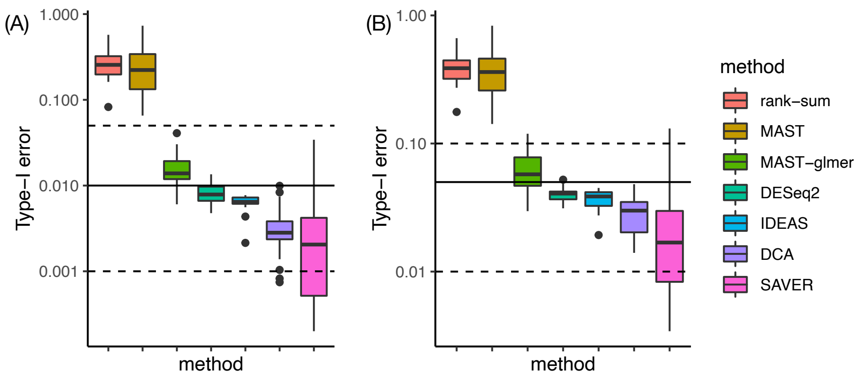

Figure S13: Type I error estimates of different methods for 17 cell types when p-value cutoff is 0.01 **(A)** or 0.05 **(B)**, respectively. The solid lines in (A) and (B) indicate the p-value cutoffs. The dash lines in (A) indicate 0.001 and 0.05, respectively. The dash lines in (B) indicate 0.01 and 0.1, respectively.

Table S3: Type I error estimation by the proportion of rejection at p-value 0.05 using permuted case/control label. This table corresponds to Supplementary Figure S13(B).

cell_type	rank-sum	MAST	MAST-glmer	DESeq2	IDEAS	DCA	SAVER
AST-FB	0.32	0.387	0.11	0.049	0.027	0.041	0.003
AST-PP	0.53	0.548	0.049	0.048	0.036	0.014	0.03
Endothelial	0.272	0.195	0.055	0.039	0.045	0.048	0.028
IN-PV	0.294	0.261	0.075	0.035	0.041	0.03	0.008
IN-SST	0.326	0.187	0.03	0.035	0.039	0.02	0.005
IN-SV2C	0.176	0.142	0.057	0.032	0.043	0.033	0.028
IN-VIP	0.332	0.259	0.047	0.037	0.043	0.035	0.039
L2_3	0.664	0.831	0.119	0.042	0.038	0.033	0.01
L4	0.539	0.46	0.067	0.041	0.037	0.028	0.008
L5_6	0.389	0.308	0.083	0.037	0.039	0.042	0.017
L5_6-CC	0.446	0.47	0.108	0.041	0.044	0.031	0.009
Microglia	0.362	0.325	0.078	0.041	0.019	0.014	0.087
Neu-mat	0.387	0.361	0.071	0.045	0.031	0.027	0.01
Neu-NRGN-I	0.277	0.21	0.047	0.031	0.042	0.039	0.007
Neu-NRGN-II	0.439	0.411	0.052	0.042	0.032	0.019	0.017
Oligodendrocytes	0.562	0.724	0.038	0.052	0.033	0.018	0.131
OPC	0.425	0.392	0.041	0.041	0.042	0.028	0.103

3.5 Overlap with SFARI genes

Table S4: Gene set enrichment analysis p-value for the enrichment of SFARI genes based on rankings of DE signals

	DESeq2	IDEAS_NB	IDEAS_DCA
PFC_IN-PV	0.09	0.27	0.18
PFC_IN-SST	0.11	0.54	0.032
PFC_IN-VIP	0.23	0.11	0.053
PFC_L2_3	0.25	0.18	0.0045
PFC_L4	0.074	0.091	0.032
PFC_L5_6	0.049	0.061	0.98

3.6 Gene set enrichment analysis (GSEA) using REACTOME pathways

We downloaded REACTOME pathway annotation `c2.cp.reactome.v7.1.symbols.gmt` from `https://data.broadinstitute.org/gsea-msigdb/msigdb/release/7.1/`, and ran GSEA using R package `fgsea`. Here we list all the results with adjusted p-value smaller than 0.05.

```
--------------------------------------------------
Endothelial
--------------------------------------------------

$DESeq2
   pathway
 1: SRP_DEPENDENT_COTRANSLATIONAL_PROTEIN_TARGETING_TO_MEMBRANE
 2: TRANSLATION
 3: RRNA_PROCESSING
 4: RESPONSE_OF_EIF2AK4_GCN2_TO_AMINO_ACID_DEFICIENCY
 5: EUKARYOTIC_TRANSLATION_INITIATION
 6: SELENOAMINO_ACID_METABOLISM
 7: EUKARYOTIC_TRANSLATION_ELONGATION
 8: REGULATION_OF_EXPRESSION_OF_SLITS_AND_ROBOS
 9: NONSENSE_MEDIATED_DECAY_NMD
10: METABOLISM_OF_AMINO_ACIDS_AND_DERIVATIVES
11: INFLUENZA_INFECTION
12: INFECTIOUS_DISEASE
13: RESPIRATORY_ELECTRON_TRANSPORT_ATP_SYNTHESIS_AND_HEAT_PRODUCTION
14: METABOLISM_OF_RNA
            pval         padj      NES
 1: 3.352074e-06 0.0005296276 1.585903
 2: 3.213129e-06 0.0005296276 1.561339
 3: 1.185720e-05 0.0009367189 1.556261
 4: 1.045400e-05 0.0009367189 1.541682
 5: 1.565277e-05 0.0009892549 1.549457
 6: 2.326745e-05 0.0012254193 1.537243
 7: 8.373781e-05 0.0037801640 1.495820
 8: 1.008269e-04 0.0039826633 1.488167
 9: 1.820414e-04 0.0063916768 1.460114
10: 2.646323e-04 0.0083623813 1.441035
11: 7.168753e-04 0.0191212959 1.411776
12: 7.261252e-04 0.0191212959 1.310402
13: 1.812653e-03 0.0409141736 1.505900
14: 1.775471e-03 0.0409141736 1.278604

$IDEAS_DCA
   pathway
1: TRANSCRIPTIONAL_REGULATION_BY_TP53
            pval        padj      NES
1:  5.480576e-06 0.001731862 1.710584

--------------------------------------------------
IN-SST
--------------------------------------------------

$DESeq2
   pathway
```

```
1: RESPIRATORY_ELECTRON_TRANSPORT
2: RESPIRATORY_ELECTRON_TRANSPORT_ATP_SYNTHESIS_AND_HEAT_PRODUCTION
3: THE_CITRIC_ACID_TCA_CYCLE_AND_RESPIRATORY_ELECTRON_TRANSPORT
4: COMPLEX_I_BIOGENESIS
5: PEPTIDE_LIGAND_BINDING_RECEPTORS
           pval        padj      NES
1: 9.246451e-07 0.000370927 1.671747
2: 1.270298e-06 0.000370927 1.616130
3: 1.706289e-05 0.003321576 1.488195
4: 2.693380e-05 0.003932335 1.695798
5: 1.469840e-04 0.017167735 1.692564

$IDEAS_NB
   pathway                    pval       padj      NES
1: L1CAM_INTERACTIONS 6.323096e-05 0.03686365 1.486866

$IDEAS_DCA
   pathway
1: RESPIRATORY_ELECTRON_TRANSPORT
2: RESPIRATORY_ELECTRON_TRANSPORT_ATP_SYNTHESIS_AND_HEAT_PRODUCTION
3: THE_CITRIC_ACID_TCA_CYCLE_AND_RESPIRATORY_ELECTRON_TRANSPORT
4: RNA_POLYMERASE_II_TRANSCRIPTION
5: COMPLEX_I_BIOGENESIS
6: NOTCH_HLH_TRANSCRIPTION_PATHWAY
           pval        padj      NES
1: 8.208884e-06 0.002554043 1.731140
2: 9.050801e-06 0.002554043 1.681495
3: 1.312008e-05 0.002554043 1.580630
4: 1.454047e-04 0.021229087 1.288993
5: 2.600301e-04 0.030371520 1.724897
6: 4.353902e-04 0.042377977 1.783170

---------------------------------------------------
L2_3
---------------------------------------------------

$IDEAS_NB
   pathway                                pval       padj       NES
1: TRANSLESION_SYNTHESIS_BY_POLK 4.693707e-05 0.03783128 -2.264327

---------------------------------------------------
L5_6
---------------------------------------------------

$IDEAS_DCA
   pathway
1: RESPIRATORY_ELECTRON_TRANSPORT
2: THE_CITRIC_ACID_TCA_CYCLE_AND_RESPIRATORY_ELECTRON_TRANSPORT
3: RESPIRATORY_ELECTRON_TRANSPORT_ATP_SYNTHESIS_AND_HEAT_PRODUCTION
4: COMPLEX_I_BIOGENESIS
           pval        padj      NES
1: 3.874828e-06 0.002968119 1.559201
2: 3.032646e-05 0.011615034 1.427667
3: 4.903206e-05 0.012519519 1.453862
```

```
4: 1.293762e-04 0.024775540 1.553722

--------------------------------------------------
L5_6-CC
--------------------------------------------------

$DESeq2
   pathway                     pval     padj     NES
1: COMPLEX_I_BIOGENESIS 2.15753e-05 0.018339 1.57771

--------------------------------------------------
Microglia
--------------------------------------------------

$DESeq2
   pathway                    pval      padj      NES
1: SIGNALING_BY_ERBB4 0.0003435272 0.0340092 1.732156

$IDEAS_DCA
   pathway                                        pval        padj      NES
 1: DEVELOPMENTAL_BIOLOGY                 2.693380e-05 0.002666446 1.563263
 2: SIGNALING_BY_ROBO_RECEPTORS           5.983003e-05 0.002961586 1.823819
 3: TOLL_LIKE_RECEPTOR_CASCADES           1.774619e-04 0.005856243 1.792809
 4: SIGNALING_BY_ERBB2                    5.092329e-04 0.010082811 1.745213
 5: NERVOUS_SYSTEM_DEVELOPMENT            4.395162e-04 0.010082811 1.559152
 6: METABOLISM_OF_RNA                     7.145970e-04 0.011790851 1.693711
 7: SIGNALING_BY_MET                      1.151892e-03 0.016291038 1.705486
 8: SIGNALING_BY_ERBB4                    1.801664e-03 0.022295587 1.681338
 9: TRANSPORT_OF_SMALL_MOLECULES          2.320680e-03 0.025527478 1.554697
10: CELLULAR_RESPONSES_TO_EXTERNAL_STIMULI  2.924422e-03 0.028951778 1.590222

--------------------------------------------------
Neu-NRGN-I
--------------------------------------------------

$DESeq2
   pathway
1: RESPIRATORY_ELECTRON_TRANSPORT
2: RESPIRATORY_ELECTRON_TRANSPORT_ATP_SYNTHESIS_AND_HEAT_PRODUCTION
3: THE_CITRIC_ACID_TCA_CYCLE_AND_RESPIRATORY_ELECTRON_TRANSPORT
4: COMPLEX_I_BIOGENESIS
5: SIGNALING_BY_MODERATE_KINASE_ACTIVITY_BRAF_MUTANTS
           pval        padj      NES
1: 2.185732e-05 0.004163820 1.568409
2: 1.819099e-05 0.004163820 1.521486
3: 5.299993e-05 0.006730991 1.466350
4: 2.922455e-04 0.027836380 1.600551
5: 6.211871e-04 0.047334456 1.665379

$IDEAS_NB
   pathway
1: RESPIRATORY_ELECTRON_TRANSPORT
2: RESPIRATORY_ELECTRON_TRANSPORT_ATP_SYNTHESIS_AND_HEAT_PRODUCTION
3: COMPLEX_I_BIOGENESIS
```

```
4: THE_CITRIC_ACID_TCA_CYCLE_AND_RESPIRATORY_ELECTRON_TRANSPORT
5: SIGNALING_BY_MODERATE_KINASE_ACTIVITY_BRAF_MUTANTS
           pval        padj      NES
1: 3.444094e-07 0.000131220 1.668845
2: 6.597622e-06 0.001256847 1.560452
3: 1.045400e-05 0.001327658 1.701401
4: 2.383151e-05 0.002269951 1.489015
5: 2.651538e-04 0.020204723 1.687284


$IDEAS_DCA
   pathway
1: RESPIRATORY_ELECTRON_TRANSPORT
2: THE_CITRIC_ACID_TCA_CYCLE_AND_RESPIRATORY_ELECTRON_TRANSPORT
3: RESPIRATORY_ELECTRON_TRANSPORT_ATP_SYNTHESIS_AND_HEAT_PRODUCTION
4: COMPLEX_I_BIOGENESIS
           pval         padj      NES
1: 1.277296e-09 2.433250e-07 1.839651
2: 6.435037e-10 2.433250e-07 1.778831
3: 4.705457e-09 5.975930e-07 1.757933
4: 1.181424e-07 1.125307e-05 1.935357

---------------------------------------------------
Oligodendrocytes
---------------------------------------------------

$IDEAS_NB
   pathway                          pval       padj      NES
1: SIGNALING_BY_RHO_GTPASES 0.0003428697 0.04731602 1.632791
```

3.7 Mean and pseudo dispersion regression

To explore what kind of differential expression patterns IDEAS_DCA captures, we compute subject-level mean and a pseudo dispersion parameter based on the denoised cell-level distributions given by DCA, and explore the associations between the log transformation of these two quantities with case/control status.

Following the notations in section 1.1 of supplementary material, for one specific gene i and a cell j, let π_{ij}, $\bar{\mu}_{ij}$ and θ_{ij} denote the corresponding elements on position (i, j) of dropout probability matrix Π, normalized mean matrix $\bar{M}$, and dispersion parameter matrix Θ respectively. The DCA-denoised distribution of gene i in cell j is

$$P_{ij} = \text{ZINB}(\pi_{ij}, \bar{\mu}_{ij}, \theta_{ij}).$$

The expectation and variance for P_{ij} are computed as

$$E(P_{ij}) = (1 - \pi_{ij})\bar{\mu}_{ij} \quad \text{and} \quad Var(P_{ij}) = (1 - \pi_{ij})\bar{\mu}_{ij}[1 + \bar{\mu}_{ij}(\pi_{ij} + 1/\theta_{ij})].$$

To simplify notation, we assume that the cells belong to a subject k occupy the first J columns of the count matrix, then the DCA-denoised distributions for the expression of this gene in the J cells are:

$$P_{i1} = \text{ZINB}(\pi_{i1}, \bar{\mu}_{i1}, \theta_{i1}),\ P_{i2} = \text{ZINB}(\pi_{i2}, \bar{\mu}_{i2}, \theta_{i2}),\ \ldots,\ P_{iJ} = \text{ZINB}(\pi_{iJ}, \bar{\mu}_{iJ}, \theta_{iJ}).$$

We treat these J distributions equally and view the cell-level expression of this gene in this subject as a random variable Y_{ik} with $1/J$ probability to be from each of the J distributions. Then the mean and

variance of Y_{ik} can be computed as

$$E(Y_{ik}) = \frac{1}{J}\sum_{j=1}^{J} E(P_{ij}) \text{ and } Var(Y_{ik}) = \frac{1}{J}\sum_{j=1}^{J} Var(P_{ij}) + \frac{1}{J}\sum_{j=1}^{J}\left[E(P_{ij}) - \frac{1}{J}\sum_{j=1}^{J} E(P_{ij})\right]^2.$$

If we assume Y_{ik} can be approximated by a negative binomial distribution, but we can compute a pseudo dispersion parameter for it as

$$disp(Y_{ik}) = \frac{[E(Y_{ik})]^2}{Var(Y_{ik}) - E(Y_{ik})}.$$

Next we can assess the association between case/control status and mean or pseudo dispersion by linear regression. Specifically, for gene i, we can assess the association between mean expression and case/control status by a linear regression with $\log[E(Y_{ik})]$ as the response variable and case/control status together with other individual level features as covariates. Similar analysis can be conducted using $\log[disp(Y_{ik})]$ as response instead as well.

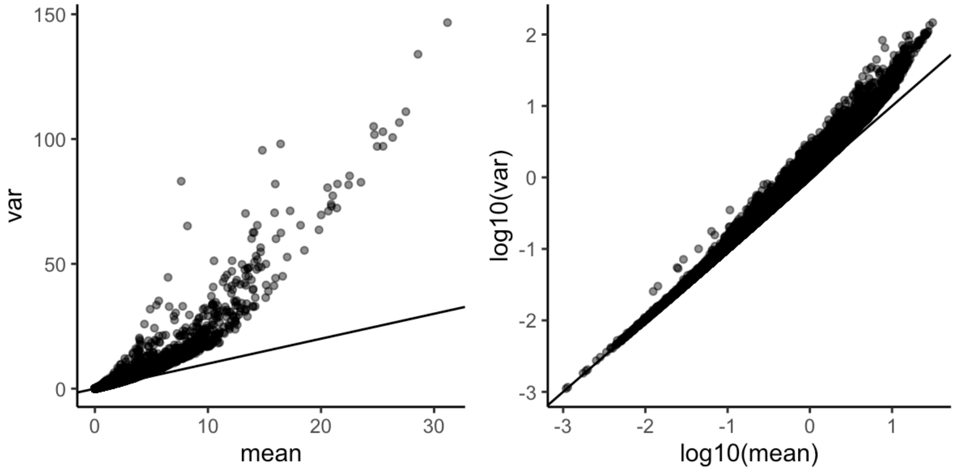

Figure S14: Compare the mean and variance for gene-specific and subject-specific distributions in the Autism data. Each of 8260 × 23 points represents one gene and one subject. 23 points having mean ≥ 35 are treated as outliers and are removed. The reference lines are $y = x$ in both figures.

Table S5: Proportion of genes with different types of DE signals. Here we claim a mean or dispersion difference if the corresponding p-value is smaller than 0.001.

Gene groups		mean diff. and dispersion diff.	mean diff. only	dispersion diff only	others
IDEAS_DCA DE	DESeq2 DE (38 genes)	0.13	0.21	0.11	0.55
	DESeq2 EE (222 genes)	0.04	0.14	0.14	0.68
IDEAS_DCA EE	DESeq2 DE (93 genes)	0	0	0.01	0.99
	DESeq2 EE (7,907 genes)	0	0	0.01	0.99

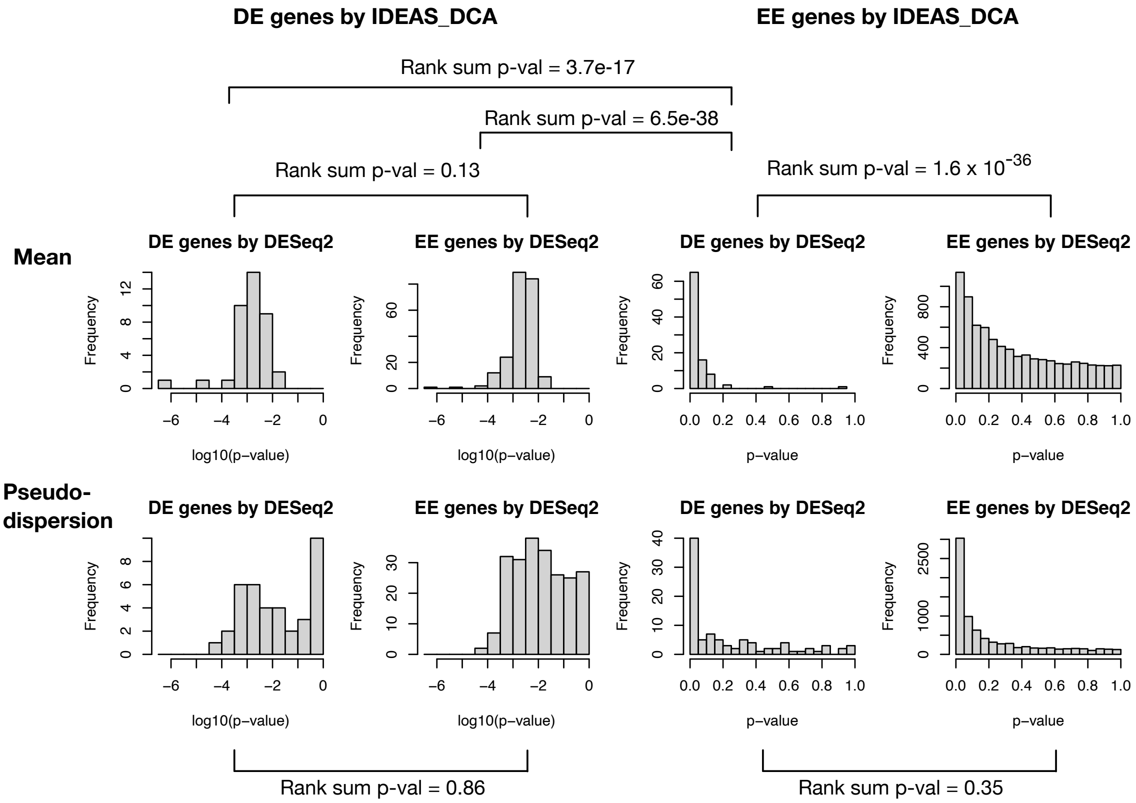

Figure S15: Compare the four groups of genes that identified as DE/EE (differentially expressed / equivalently expressed) by IDEAS_DCA or DESeq2 : 38 genes identified as DE genes by both methods, 222 and 93 genes identified by IDEAS_DCA and DESeq2 only, respectively, and the remaining 7907 genes. The first / second row shows the p-value (or log10(p-value) distribution when comparing mean values / pseudo-dispersion from DCA-denoised scRNA-seq data.

4 Supplementary results from COVID-19 data analysis

4.1 Data processing and DE results

We studied a single-cell RNA-seq data set of COVID-19 patients to assess gene expression difference between patients with mild and severe symptoms. The data set we used was collected from blood samples (cohort 1 of Schulte-Schrepping et al. 2020) using 10x Chromium platform. It contains normalized expression of 46,584 genes in 99,049 cells, and can be downloaded from link `https://beta.fastgenomics.org/datasets/detail-dataset-952687f71ef34322a850553c4a24e82e`. We recovered the RNA-seq counts from the normalized expression by taking exponential, subtracting 1, dividing by 10,000 and multiplying by total UMI counts. 10,176 genes were left after genes appearing in less than 2,000 cells (about 2%) were filtered out. We focused on cell type CD8+ T cells_1 (cluster 1 of CD8+ T cells) in COVID patients and filtered out the patients with less than 10 cells in this this cluster. After filtering, the dataset includes 5,557 cells from 17 COVID patients (10 severe cases vs. 7 mild ones). Altogether 5,160 genes expressed in at least 90% of the 5,557 cells were kept for analysis.

The current version of DCA code from `https://github.com/theislab/dca` (accessed on 11/02/2021) no longer provides the normalized mean matrix $\bar{M}$ as one of the output files. But $\bar{M}$ can be retrieved based on the mean matrix M from the output and the original count matrix. The code for this step of processing on COVID data is included in our github repo: `https://github.com/Sun-lab/ideas_pipeline/tree/main/COVID`.

4.2 Type I error evaluation

Similar to the analysis of Autism data, we permuted case/control labels and then calculated the proportion of DE genes at different p-value cutoffs to evaluate type I error. For Autism data, we considered multiple cell types and thus summarized the type I error across cell types by a box plot. Here we only considered one cell type and we listed the type I error at different p-value cutoffs. At p-value cutoff 0.01 or 0.05, MAST_glmer has much higher type I error than in Autism data or simulation, likely because smaller sample size and more uneven number of cells per subject.

Table S6: The estimates of type I error for different methods (rows) at different p-value cutoffs (columns). The p-values of IDEAS methods, including IDEAS_DCA and IDEAS_SAVER were calculated by up to 99,999 permutations.

methods	1e-04	0.001	0.01	0.05
rank-sum	0.18	0.24	0.35	0.47
MAST	0.38	0.52	0.71	0.85
MAST-glmer	0.0062	0.043	0.2	0.47
DESeq2	0	0.0014	0.006	0.026
IDEAS	0	0.00058	0.014	0.085
IDEAS_DCA	0	0	0.00078	0.044
IDEAS_SAVER	0	0.00019	0.0074	0.073

4.3 GSEA using gene mean expression level

To explore the possible influence of gene expression level on the pathways identified by different methods in the COVID-19 data set, we compute a mean expression level for each gene, rank genes by this quantity, and carry out gene set enrichment analysis.

To compute the mean expression level, we first rescale the counts in the pseudo bulk count matrix to adjust for subject-level read depth. For a pseudo bulk count matrix K of size (m, n), with each row corresponding to a gene and each column corresponding to a subject, the rescaled counts are computed by

$$R_{ij} = \frac{K_{ij}}{s_j} \quad \text{with} \quad s_j = \frac{\sum_{i=1}^{m} K_{ij}}{\underset{j \in 1,\ldots,n}{\text{median}} \sum_{i=1}^{m} K_{ij}}.$$

After rescaling, the mean expression level of the i-th gene is computed as $\sum_{j=1}^{n} R_{ij}/n$ and it is used as the gene-level statistic for gene set enrichment analysis. On this COVID-19 data set, at adjusted p-value cutoff 0.05, GSEA using mean expression level identified 235 pathways.

4.4 GSEA results

The GSEA results from all the methods were included in Additional File 2. Here we illustrate the results based on the gene rankings by IDEAS_DCA and IDEAS_SAVER.

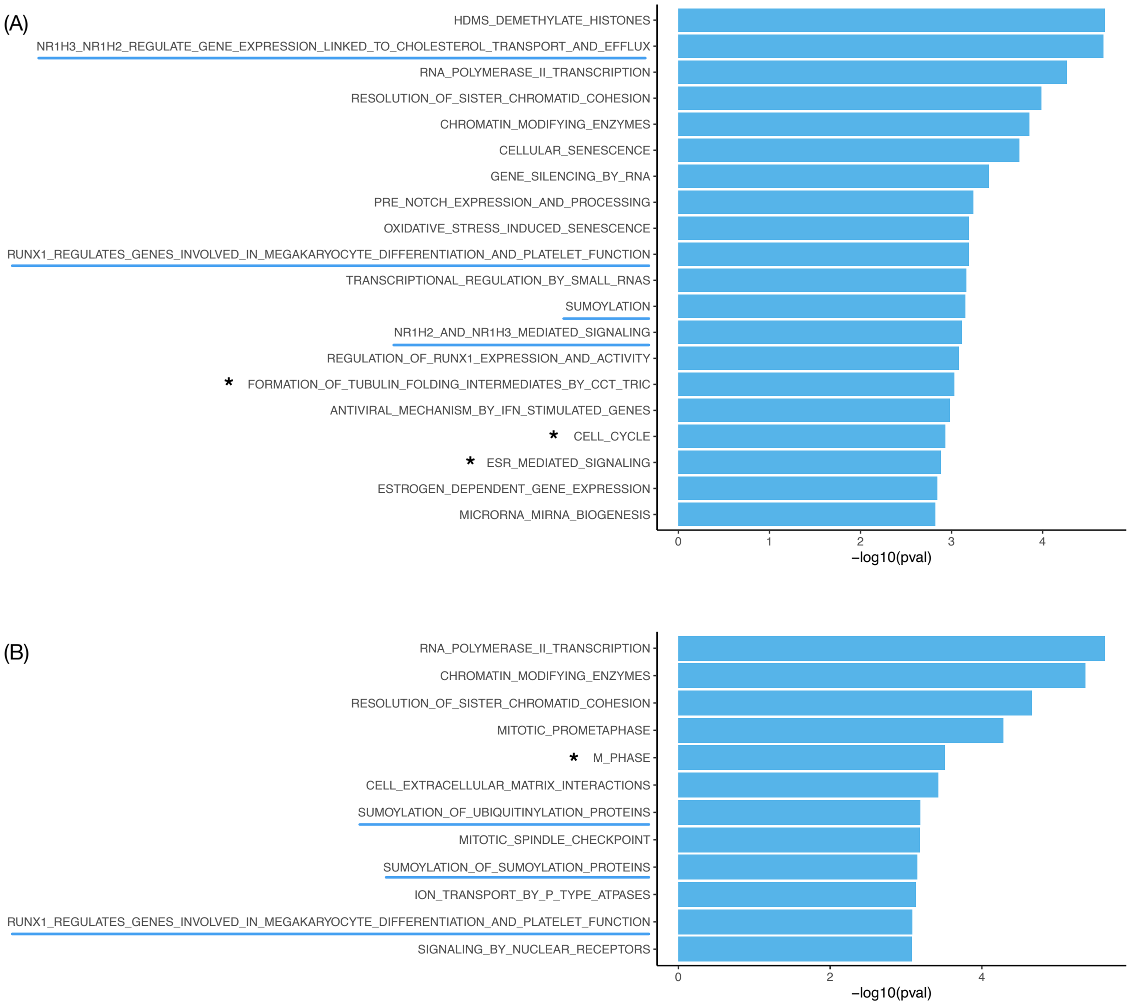

Figure S16: GSEA results based on the gene rankings by IDEAS_DCA (A) and IDEAS_SAVER(B). All these pathways have adjusted p-value smaller than 0.05. The underscored pathways are those mentioned in the main text, and the ones with * to the left are those that are also identified by GSEA analysis if we rank genes by gene expression level.
