# Supplementary material for: IDEAS: individual level differential expression analysis for single-cell RNA-seq data
Source: Genome Biol. 2022 Jan 24;23:33. doi: 10.1186/s13059-022-02605-1 (PMC8784862; doi:10.1186/s13059-022-02605-1)
Supplement: Supplementary file 3 — Additional file 3 Review history. [file 13059_2022_2605_MOESM3_ESM.docx]

Review History

**First round of review**

**Reviewer 1**

**Were you able to assess all statistics in the manuscript, including the appropriateness of statistical tests used?**

Yes: The statistics is solid.

**Were you able to directly test the methods?**

No

**Comments to author:**

Reviewer comment for “Individual Level Differential Expression Analysis for Single Cell RNA-seq data”

In this work, the authors presented “IDEAS”, which was developed to assess the difference of a certain cell type in a case-and-control study design. Compared with existing approaches, including (pseudo-bulk + Deseq2), Wilcoxon rank sum test, and MAST, IDEAS can control type I error (Wilcoxon rank sum test and MAST cannot control type I error), and have good power for detecting differences in the variance (DEseq2 is comparable to IDEAs in the inference of the difference in mean, but has less power in detecting the difference in the variance). The results in the work look solid. The overall idea is novel to the field of single-cell genomics. Here are my comments:

1. Scope of the proposed method. Based on the method description and examples, the proposed method focuses on case-and-control design: it compares a certain cell type (e.g. microglia cells) between case (e.g. autistic patients) and control (e.g. normal). I would like the authors to clarify whether the proposed method is restricted to this study design. Another common setting that researchers encounter in practice is like this: scRNA-seq are collected from the tumour tissues in multiple cancer patients, and the question of interest is to compare cell type A and cell type B across patients. Can IDEAS be applied to this setting?

2. Other than MAST (Genome Biology, 2015), it is good to compare IDEAS with other more recent methods developed for scRNA-seq differential expression analysis, including:

• Keegan D Korthauer, Li-Fang Chu, Michael A Newton, Yuan Li, James Thomson, Ron Stewart, Christina Kendziorski. A statistical approach for identifying differential distributions in single-cell RNA-seq experiments. Genome Biology, 17:222. doi: 10.1186/s13059-016-1077-y

• Risso D, Perraudeau F, Gribkova S, Dudoit S, Vert JP. A general and flexible method for signal extraction from single-cell RNA-seq data. Nat Commun. 2018; 9(1):1–17.

3. It will be good to present computational cost of the proposed method.

4. For the simulation study, page 3 line 34, “varDE, 1.5 fold change”, does this refer to the change in the dispersion parameter?

5. One real example is presented comparing autistic patients and normal people. It will be good to present another real data example from a different biological tissue.

6. To estimate the individual level distribution, in page 9 line 34, “Therefore, we perform ZINB regression of confounding factors such as the cell-level read depth”. It will be good to provide more technical detail (how exactly are parameters in ZINB distribution determined in this regression-based approach) on how this is implemented.

7. The link (https://github.com/Sun-lab/ideas) to the software gives an error and cannot be accessed on the day that I tried.

Minor comments

8. Page 2 line 13, “when sample size is large”: here sample size refers to the number of individuals. It is better to use the number of individuals instead of sample size, for easier understanding, and not to confuse with the number of cells.

**Reviewer 2**

**Were you able to assess all statistics in the manuscript, including the appropriateness of statistical tests used?**

Yes: All of the statistical methods are detailed described in the manuscript. However, I just could not access the code and thus unable to directly test the tool, check the implementation or the reproducibility.

**Were you able to directly test the methods?**

No

**Comments to author:**

Zhang et al. describe a statistical method, named IDEAS, which assesses differential expression in scRNA-seq data, through directly comparing the gene expression distributions across individuals. IDEAS first estimates the expression distribution per gene per individual after raw count denoising, and further tests the DE by a distance based test or kernel regression based test. Overall the authors have mostly thoroughly explained their approach and model. The model is quite important in scRNA-seq analysis as DE across conditions is an essential step in the general pipeline. However, please consider the following comments and questions.

Major

1. Most of the statistical methods are detailed described in the manuscript. However, I could not access the code from the Github link and thus unable to test the tool, check the implementation or the reproducibility.

2. The authors tested the performance of IDEAS by simulation datasets, which is very beneficial to the readers. However, it lacks a systematic comparison, to name a few:

a. The authors set meanDE 1.2 fold and varDE 1.5 fold, what is the relationship between different fold changes vs type I error and power as well as other methods in comparisons?

b. In the 5 case vs 5 control, the authors sampled 1080 cells per subject, but 360 cells per subject in 13 cases vs 10 controls. Can the author explain why? Shall we control the number of cells and only test the numbers of sample variables and vice versa?

c. Related to b, how does # of cases vs controls, imbalance cases vs controls, and # of cells per sample affect power and type I error? This information can help the readers understand the advantages and limitations of IDEAS.

3. The authors compared IDEAS performance based on type I error and power separately. As there are win and loss between IDEAS and other methods, could we compare the performance with a combined statistics such as F-score?

4. Could we describe how we do the multiple testing corrections? Is it a major concern for IDEAS?

5. Computational intensity is the main concern for me for IDEAS, especially compared to the current state of the art methods, as IDEAS is based on a few permutation test . I wonder if we could compare the CPU time (or User time) across different methods? Is multi-core / parallel implementation available for IDEAS? Any guideline would help the users' decision on analysis package selection.

Minor

1. Could we add more details in Figure 1's descriptions. Such as number of individuals in case and control etc.

2. Missing X axis of figure 3 c & d

3. Incorrect reference: Supplement 2.2 in Data denoising before DE testing section. Do you mean Supplement 3.2?

4. All gene names should be italicized. E.g. SLC4A8

5. Incorrect reference: Supplement 2.5 in Differentially expressed pathways in microglia section.

6. Typo in KL divergence in method section: DKL(P||M): f_Q -> f_M

**Reviewer 3**

**Were you able to assess all statistics in the manuscript, including the appropriateness of statistical tests used?**

Yes: This paper is statistically rigorous and novel. The statistical novelty is the strength of this paper.

**Were you able to directly test the methods?**

Yes

**Comments to author:**

This paper proposes a novel method for analysis of multi-subject single cell data, where single cell RNA sequencing is performed across multiple subjects, coming from different cohorts (eg case, control). This study design is now becoming very common as single cell experiments are now broadly applied at the population level in the study of disease. The authors study, specifically, the problem of differential expression (DE) analysis, i.e. finding genes that have different expression across different groups of subjects. Most existing methods for differential expression analysis are either (1) for bulk RNA-seq, or (2) for single cell RNA-seq, but ignoring subject-to-subject variation within a group. Thus, this study is unique in proposing a DE method that is uniquely designed for multi-subject single cell data, taking into consideration both inter-subject as well as inter-cell variation. Thus, I think it fills a critical analysis gap in the literature.

The idea proposed is to first estimate the gene expression distribution within each subject, and then, comparing these distributions across subjects and seeing whether they differ more between groups as compared to within groups. This way, they can find DE genes that differ not only in mean across groups, but also in other aspects of the distribution, such as variance. This allows their method to find DE signals that are missed by existing methods.

I think the work is significant and the paper is mostly well written. However, I have some concerns/questions:

1. It took me a while to figure out what the paper is doing. In abstract: "Applying such methods to assess individual-level difference can lead to inflated type 1 error." -- I have no idea wht "individual-level difference" means. The abstract and intro keeps going and uses "individual-level differential expression" to describe their work, but I think many readers will be confused as to what that refers to. I think the authors can take more care to set up the problem clearly, and give its background in the many disease-related studies using scRNA-seq, or the single cell eQTLgen consortium. Perhaps also lay the groundwork about what the term "differential expression" could mean, if we have single cell level data across multiple subjects in each group. Especially for readers not familiar with Statistics, this is necessary.

2. While it is true that the method, as designed, captures any difference in distribution across groups, how would one interpret and follow-up on the results? When the difference is not a mean-level shift, is there a way to characterize it in a way that gives us a sense of what about the distribution is changed? While such omnibus tests are good in that they capture everything, I find that the results are often harder to interpret.

3. It is interesting that you find denoising to improve results. The difference between SAVER and DCA is that SAVER adds a Bayesian shrinkage step on top of the manifold fit. But neither SAVER nor DCA, as is, provides a good estimate of the underlying gene expression distribution, as the denoising step removes biological as well as technical variance. However, SAVER allows for a post-denoising sampling step, where instead of taking the posterior mean, the gene expression for each cell is sampled from the posterior distribution. This mitigates the denoising bias and gives an unbiased estimate of the underlying distribution (for example, it recovers the true variances and covariances). See Agarwal et al. Statist. Sci. 35(1): 112-128 (February 2020). DOI: 10.1214/19-STS7560. Thus, should you apply this posterior sampling step if you were to use denoising methods?

4. How do the IDEAS genes differ from those that are found by, say, DESeq2? I am guessing that the set of genes significant for IDEAS but not for DESeq2 are those that don't have a bulk shift in mean, but are more variable/bursty or have changes in other aspects of their distribution? Or, is it simply a "denoising" effect in that this new test allows more sensitive detection of subtle changes in mean? Are there instances where we can give such distributional changes a biological interpretation?

**Authors Response**

**Point-by-point responses to the reviewers’ comments:**

Response to Reviewer comment for “Individual Level Differential Expression Analysis for Single Cell RNA-seq data”

*We appreciate all the reviewers’ time and effort to review our paper and give many valuable and constructive comments and suggestions. We have added point-to-point response below and our response is in blue color. We have also highlighted some edits in the main text by blue color for your convenience to track updates.*

Reviewer 1

In this work, the authors presented “IDEAS”, which was developed to assess the differenc of a certain cell type in a case-and-control study design. Compared with existing approaches, including (pseudo-bulk + Deseq2), Wilcoxon rank sum test, and MAST, IDEAS can control type I error (Wilcoxon rank sum test and MAST cannot control type I error), and have good power for detecting differences in the variance (DEseq2 is comparable to IDEAs in the inference of the difference in mean, but has less power in detecting the difference in the variance). The results in the work look solid. The overall idea is novel to the field of single-cell genomics. Here are my comments:

1. Scope of the proposed method. Based on the method description and examples, the proposed method focuses on case-and-control design: it compares a certain cell type (e.g. microglia cells) between case (e.g. autistic patients) and control (e.g. normal). I would like the authors to clarify whether the proposed method is restricted to this study design. Another common setting that researchers encounter in practice is like this: scRNA-seq are collected from the tumour tissues in multiple cancer patients, and the question of interest is to compare cell type A and cell type B across patients. Can IDEAS be applied to this setting?

*Thank you. This is a great point. IDEAS focuses on the setting to compare one cell type between cases and controls, and it should not be applied to the setting to compare cell type A and B across patients. The latter is a very interesting problem of hypothesis testing with paired samples, though currently IDEAS cannot handle this paired comparison problem. We have clarified this in the revised manuscript (the last paragraph of Background Section, at the beginning of page 2).*

2. Other than MAST (Genome Biology, 2015), it is good to compare IDEAS with other more recent methods developed for scRNA-seq differential expression analysis, including:

• Keegan D Korthauer, Li-Fang Chu, Michael A Newton, Yuan Li, James Thomson, Ron Stewart, Christina Kendziorski. A statistical approach for identifying differential distributions in single-cell RNA-seq experiments. Genome Biology, 17:222. doi: 10.1186/s13059-016-1077-y

• Risso D, Perraudeau F, Gribkova S, Dudoit S, Vert JP. A general and flexible method for signal extraction from single-cell RNA-seq data. Nat Commun. 2018; 9(1):1–17.

*Thank you for your suggestion. We have included the comparison to scDD (Korthauer et al. 2016) and ZINB-WaVE (Risso et al. 2018) in the revised manuscript (Figure 2 and supplementary Figures in Section 2.2 of Supplementary Materials). ZINB-WaVE itself is designed to characterize the distribution of scRNA-seq data and identify the corresponding low dimensional representation. A later extension by Van den Berge et al. (2018) shows that ZINB-WaVE can be used to generate gene- and cell-specific weights: the posterior probabilities that a count was generated from the NB count component instead of the zero-inflated component. Then given such weights, one can apply a bulk DE-analysis pipeline, such as DESeq2, to scRNA-seq data. We have implemented this approach to perform DE analysis using ZINB-WaVE. The simulation results show that both scDD and ZINB-WaVE have inflated type I error, like other methods designed to compare two groups of cells. This is expected since they are designed to compare two groups of cells instead of two groups of subjects.*

3. It will be good to present computational cost of the proposed method.

*Thank you, we have added the computational cost of IDEAS at the end of Discussion section.*

4. For the simulation study, page 3 line 34, “varDE, 1.5 fold change”, does this refer to the change in the dispersion parameter?

*No, it is the fold change of variance rather than dispersion. We adjusted dispersion so that the fold change of variance is 1.5 fold.*

5. One real example is presented comparing autistic patients and normal people. It will be good to present another real data example from a different biological tissue.

*Thank you for your suggestion. We have included another real data example comparing gene expression of COVID-19 patients with severe vs. mild outcomes. Overall, the results are similar to Autism data analysis in that IDEAS combined with DCA or SAVER has higher power. Our analysis identified a few pathways (by gene set enrichment analysis) that are closely related with COVID-19. For example, we identified an NR1H2 related pathway and a recent GWAS study identified a locus in NR1H2 associated with severe COVID cases. We also identified a RUNX1 related pathway, and a recent study suggested that RUNX1 inhibitor may be beneficial as both a treatment and preventive therapy for COVID-19. We have added these results in the last sub-section of Results section (page 8 to page 9).*

6. To estimate the individual level distribution, in page 9 line 34, “Therefore, we perform ZINB regression of confounding factors such as the cell-level read depth”. It will be good to provide more technical detail (how exactly are parameters in ZINB distribution determined in this regression-based approach) on how this is implemented.

*We have used the zeroinfl function from R package pscl to perform the ZINB regression. We have clarified this in the revised manuscript (page 12).*

7. The link (https://github.com/Sun-lab/ideas) to the software gives an error and cannot be accessed on the day that I tried.

*I apologize for this. It has been fixed now. This link https://github.com/Sun-lab/ideas is our R package and all the pipelines are shared at https://github.com/Sun-lab/ideas_pipeline/.*

Minor comments

8. Page 2 line 13, “when sample size is large”: here sample size refers to the number of individuals. It is better to use the number of individuals instead of sample size, for easier understanding, and not to confuse with the number of cells.

*Thanks, we have changed it.*

Reviewer 2:

Zhang et al. describe a statistical method, named IDEAS, which assesses differential expression in scRNA-seq data, through directly comparing the gene expression distributions across individuals. IDEAS first estimates the expression distribution per gene per individual after raw count denoising, and further tests the DE by a distance based test or kernel regression based test. Overall the authors have mostly thoroughly explained their approach and model. The model is quite important in scRNA-seq analysis as DE across conditions is an essential step in the general pipeline. However, please consider the following comments and questions.

Major

1. Most of the statistical methods are detailed described in the manuscript. However, I could not access the code from the Github link and thus unable to test the tool, check the implementation or the reproducibility.

*I apologize for this. It has been fixed now. This link https://github.com/Sun-lab/ideas is our R package and all the pipelines are shared at https://github.com/Sun-lab/ideas_pipeline/.*

2. The authors tested the performance of IDEAS by simulation datasets, which is very beneficial to the readers. However, it lacks a systematic comparison, to name a few:

a. The authors set meanDE 1.2 fold and varDE 1.5 fold, what is the relationship between different fold changes vs type I error and power as well as other methods in comparisons?

*Thank you for your suggestion. In the revised manuscript (Supplementary Figures 8 and 9), we have included the comparison at other fold changes and showed the type I error stays constant for different fold changes while power increases with fold changes.*

b. In the 5 case vs 5 control, the authors sampled 1080 cells per subject, but 360 cells per subject in 13 cases vs 10 controls. Can the author explain why? Shall we control the number of cells and only test the numbers of sample variables and vice versa?

*Sorry that we did not clarify this choice. It was because the powers of all the methods are very low when sample size is 5 vs. 5 and the number of cells per subject is 360, and both 360 and 1080 are reasonable number of cells per subject in real data analysis. We have mentioned this in the revised manuscript (page 3, the 1st paragraph in section “IDEAS can identify more DE patterns than the pseudo-bulk method”) and included additional results of different sample sizes (5 vs. 5, 10 vs. 10, 13 vs. 10, and 20 vs. 20) and the number of cells per subject (360 vs. 1080) in Section 2.2 and 2.3 of the supplementary materials.*

c. Related to b, how does # of cases vs controls, imbalance cases vs controls, and # of cells per sample affect power and type I error? This information can help the readers understand the advantages and limitations of IDEAS.

*Thank you. We agree such summary are very helpful. Briefly, increasing the number of cells per subject or the number of subjects both improve the power while the latter is more helpful. We have the complete set of simulation results in Section 2 of supplementary materials. We have tested imbalance of cases vs. controls (e.g., 13 cases vs. 10 controls), because that is the sample size in the Autism data that we have analyzed. In the revised paper, we have also added the results with sample size 10 vs. 10. Imbalance do not affect type I error since we use permutations to evaluate significance. Though severe imbalance (e.g., 3 vs. 17) does limit the number of possible permutation and thus limits the accuracy to evaluate p-values by permutations. We have added summary of these results in revised manuscript (3rd paragraph at page 4).*

3. The authors compared IDEAS performance based on type I error and power separately. As there are win and loss between IDEAS and other methods, could we compare the performance with a combined statistics such as F-score?

*Thank you for your suggestion. We agree that F-score is a reasonable alternative. However, in practice, we would expect a hypothesis testing method controls type I error. If a method does not control type I error, even if it has very high power, it is not desirable because it is hard to interpret the results. Therefore, we still prefer to compare power among the methods that can control type I error.*

4. Could we describe how we do the multiple testing corrections? Is it a major concern for IDEAS?

*Multiple testing corrections are done by controlling q-value. We have highlighted at page 6 of the revised manuscript. It is the same procedure for all the methods, and it is not a concern for IDEAS. For each method, we take the gene-specific p-values and use them as the input to evaluate q-values.*

5. Computational intensity is the main concern for me for IDEAS, especially compared to the current state of the art methods, as IDEAS is based on a few permutation test . I wonder if we could compare the CPU time (or User time) across different methods? Is multi-core / parallel implementation available for IDEAS? Any guideline would help the users' decision on analysis package selection.

*Yes, of course. Parallel computation is available for IDEAS. In the revised manuscript, we have summarized the computational time at the end of paper. The permutation test of IDEAS is fast enough for large scale computations. This is because when doing permutations, we just need to permute the distance matrix across all subjects and then calculating test statistic based on the distance matrix is relatively simple.*

Minor

1. Could we add more details in Figure 1's descriptions. Such as number of individuals in case and control etc.

*Yes, we have added more description for Figure 1.*

2. Missing X axis of figure 3 c & d

*Thanks a lot! We have added the x-axis labels.*

3. Incorrect reference: Supplement 2.2 in Data denoising before DE testing section. Do you mean Supplement 3.2?

*Yes, it should be Supplementary Materials Section 3.2. Thank you! We have fixed it.*

4. All gene names should be italicized. E.g. SLC4A8

*Thank you! We have fixed it.*

5. Incorrect reference: Supplement 2.5 in Differentially expressed pathways in microglia section.

*Sorry about it and thanks a lot for pointing this out. We have fixed it.*

6. Typo in KL divergence in method section: DKL(P||M): f_Q -> f_M

*Thank you! We have fixed it.*

Reviewer #3: This paper proposes a novel method for analysis of multi-subject single cell data, where single cell RNA sequencing is performed across multiple subjects, coming from different cohorts (eg case, control). This study design is now becoming very common as single cell experiments are now broadly applied at the population level in the study of disease. The authors study, specifically, the problem of differential expression (DE) analysis, i.e. finding genes that have different expression across different groups of subjects. Most existing methods for differential expression analysis are either (1) for bulk RNA-seq, or (2) for single cell RNA-seq, but ignoring subject-to-subject variation within a group. Thus, this study is unique in proposing a DE method that is uniquely designed for multi-subject single cell data, taking into consideration both inter-subject as well as inter-cell variation. Thus, I think it fills a critical analysis gap in the literature.

The idea proposed is to first estimate the gene expression distribution within each subject, and then, comparing these distributions across subjects and seeing whether they differ more between groups as compared to within groups. This way, they can find DE genes that differ not only in mean across groups, but also in other aspects of the distribution, such as variance. This allows their method to find DE signals that are missed by existing methods.

I think the work is significant and the paper is mostly well written. However, I have some concerns/questions:

1. It took me a while to figure out what the paper is doing. In abstract: "Applying such methods to assess individual-level difference can lead to inflated type 1 error." -- I have no idea what "individual-level difference" means. The abstract and intro keeps going and uses "individual-level differential expression" to describe their work, but I think many readers will be confused as to what that refers to. I think the authors can take more care to set up the problem clearly, and give its background in the many disease-related studies using scRNA-seq, or the single cell eQTLgen consortium. Perhaps also lay the groundwork about what the term "differential expression" could mean, if we have single cell level data across multiple subjects in each group. Especially for readers not familiar with Statistics, this is necessary.

*Sorry for the confusion. We have completely re-written the abstract to emphasize that we aim to detect gene expression difference between two groups of subjects. We also modified the introduction section to clarify the application setting of our method.*

2. While it is true that the method, as designed, captures any difference in distribution across groups, how would one interpret and follow-up on the results? When the difference is not a mean-level shift, is there a way to characterize it in a way that gives us a sense of what about the distribution is changed? While such omnibus tests are good in that they capture everything, I find that the results are often harder to interpret.

*Thank you for your comments. We totally agree that it is crucial to characterize what aspect of the gene expression distribution is different. At first, we sought to evaluate variance difference, but realized that there is a very strong association between mean and variance, which confounds the results (Supplementary Figure 14). Therefore, we derived a pseudo dispersion parameter estimate (Section 3.7 of Supplementary Materials) by assuming for the subject level distribution variance = mean + mean^2/disp, i.e., the mean-variance relation of a negative binomial. Then disp = mean^2/(variance - mean). We found that those genes that were identified by IDEAS but missed by DESeq2 were more likely to have differential dispersion without mean-shift than those genes identified by both methods. Though the difference was not very large, which might be partly because this dispersion parameter estimate was inaccurate. Even if it was accurate, it could only capture part of the difference of two distributions. We added a section in the revised manuscript (page 8) to discuss this additional comparison. We also mentioned in the Discussion section that this is a direction that warrants more studies: “It is important to clarify what aspects of the gene expression distributions contribute to DE signals. Though due to the sparsity of the scRNA-seq data, it is hard to quantify gene expression difference in higher moments. This is an important direction for future research”*

3. It is interesting that you find denoising to improve results. The difference between SAVER and DCA is that SAVER adds a Bayesian shrinkage step on top of the manifold fit. But neither SAVER nor DCA, as is, provides a good estimate of the underlying gene expression distribution, as the denoising step removes biological as well as technical variance. However, SAVER allows for a post-denoising sampling step, where instead of taking the posterior mean, the gene expression for each cell is sampled from the posterior distribution. This mitigates the denoising bias and gives an unbiased estimate of the underlying distribution (for example, it recovers the true variances and covariances). See Agarwal et al. Statist. Sci. 35(1): 112-128 (February 2020). DOI: 10.1214/19-STS7560. Thus, should you apply this posterior sampling step if you were to use denoising methods?

*Thank you for your suggestion. We indeed seek to sample from the posterior distribution instead of taking the posterior mean. Though in our implementation, to improve computational efficiency, we directly add up the cell level densities to derive the subject level density, instead of sampling from the cell level distributions. The results of two approaches (direct summation vs. sampling) are similar. We have clarified this point in the revised manuscript (page 6, the paragraph before “IDEAS combined with denoising improves the power to identify DE genes”). It is a great point that denoising could remove both biological and technical variance and thus it should be used with caution. We have added this point in the Discussion section.*

4. How do the IDEAS genes differ from those that are found by, say, DESeq2? I am guessing that the set of genes significant for IDEAS but not for DESeq2 are those that don't have a bulk shift in mean, but are more variable/bursty or have changes in other aspects of their distribution? Or, is it simply a "denoising" effect in that this new test allows more sensitive detection of subtle changes in mean? Are there instances where we can give such distributional changes a biological interpretation?

*Thank you for these great questions. We have added a section in the revised manuscript “Both denoising and non-mean-shift signals contribute to the improved power of IDEAS”. As mentioned earlier to answer your question 2, we have introduced a new quantity of (pseudo) dispersion and compare it between cases and controls. We found those genes identified by IDEAS but missed by DESeq2 are more likely to have DE signals only by dispersion (Supplementary Table 5). Though the difference is not very large. It warrants future work to better characterize the distribution difference and the associated biological interpretation.*

**Second round of review**

**Reviewer 1**

All my previous comments have been addressed.

**Reviewer 2**

The authors have addressed all my previous concerns on the manuscript. There are a few minor issues 1. I couldn't install the ideas package followed the readme on the github page. I suspect that the name of the branch has to be "master" rather than "main" for devtools to recognize the package automatically. 2. It would be very helpful for the users if the authors can provide a package reference (maybe in pdf or a markdown) rather than read through the Rd files in man/. Specifically what are the required inputs for each function and descriptions of individual functions.

**Reviewer 3**

The authors have addressed all of my concerns, and I don’t have additional ones. I would support the publication of this paper.

**Authors Response**

**Point-by-point responses to the reviewers’ comments:**

Reviewer #2: The authors have addressed all my previous concerns on the manuscript. There are a few minor issues

1. I couldn't install the ideas package followed the readme on the github page. I suspect that the name of the branch has to be "master" rather than "main" for devtools to recognize the package automatically.

*Three of the co-authors have tried to install the package and it works for all three of us. The change from "master" to "main" is a systematic change of GitHub to all repositories. It might cause temporary issues and has been resolved now.*

2. It would be very helpful for the users if the authors can provide a package reference (maybe in pdf or a markdown) rather than read through the Rd files in man/. Specifically what are the required inputs for each function and descriptions of individual functions.

*We have provided a package reference and toy example with markdown format at https://github.com/Sun-lab/ideas*
